# An integrated body composition– immunonutritional signature for predicting immunotherapy response and prognosis in gastric cancer: a multicenter retrospective cohort study

**DOI:** 10.3389/fonc.2026.1875482

**Published:** 2026-07-15

**Authors:** Fang Li, Tao Zheng, Honghai Guo, Peigang Yang, Ning Meng, Xiaolong Li, Zhenjiang Guo, Yuan Tian, Qun Zhao

**Affiliations:** 1 Department of Pathology, The Fourth Hospital of Hebei Medical University, Shijiazhuang, Hebei, China; 2 The Third Department of Surgery, The Fourth Hospital of Hebei Medical University, Shijiazhuang, Hebei, China; 3 Hebei Key Laboratory of Precision Diagnosis and Comprehensive Treatment of Gastric Cancer, Shijiazhuang, Hebei, China; 4 Big Data Analysis and Mining Application for Precise Diagnosis and Treatment of Gastric Cancer, Hebei Provincial Engineering Research Center, Shijiazhuang, Hebei, China; 5 Department of General Surgery, Shijiazhuang People’s Hospital, Shijiazhuang, Hebei, China; 6 Department of General Surgery, Baoding Central Hospital, Baoding, Hebei, China; 7 Department of General Surgery, Hengshui People’s Hospital, Hengshui, Hebei, China

**Keywords:** body composition, disease-free survival, immunonutritional signature, locally advanced esophagogastric adenocarcinoma, neoadjuvant immunochemotherapy, pathological complete response

## Abstract

**Background:**

Response to neoadjuvant PD-1 inhibitor plus chemotherapy in locally advanced esophagogastric adenocarcinoma varies widely, and tumor biomarkers alone do not fully explain the variation. We developed an integrated Body Composition and Immunonutritional Signature (BCIS) from pretreatment CT body composition and routine blood markers, and tested whether it predicts pathological response, immune-related adverse events (irAEs), and survival.

**Methods:**

From four tertiary hospitals in Hebei Province, China, we enrolled 720 patients with histologically confirmed gastric or gastroesophageal junction (Siewert II/III) adenocarcinoma treated between 2019 and 2023 with neoadjuvant PD-1 inhibitor plus SOX or XELOX followed by D2 gastrectomy. BCIS was a 0–10 additive score from ten prespecified adverse host features. Analyses included logistic regression, restricted cubic spline (RCS) modeling, multivariable Cox regression, DeLong tests for nested AUC comparisons, decision-curve analysis, eleven sensitivity scenarios, and collinearity checks by Spearman correlation and variance inflation factors.

**Results:**

BCIS classified 243 (33.8%) patients as favorable, 303 (42.1%) as intermediate, and 174 (24.2%) as unfavorable. Major pathological response (MPR) rates dropped sharply across strata (64.2%, 39.6%, 21.8%; P-trend<0.001). After adjusting for age, sex, cT/cN, PD-L1 CPS, MMR status, EGJ origin, and regimen, every BCIS point cut the odds of MPR by roughly 40% (adjusted OR = 0.61, 95% CI 0.55–0.68, P<0.001) and pCR by close to half (adjusted OR = 0.55, 95% CI 0.46–0.66, P<0.001). RCS modeling flagged CRP and CAR as nonlinear; BCIS itself was strictly linear. Adding BCIS to a clinical baseline lifted overall AUC for MPR from 0.597 to 0.710 (DeLong P<0.001) and external AUC from 0.588 to 0.704. At 42.5 months’ median follow-up, each BCIS point raised the hazard of progression by 53% (adjusted HR = 1.53, 95% CI 1.44–1.63) and of death by 38% (adjusted HR = 1.38, 95% CI 1.29–1.47; both P<0.001). Direction of effect held across every prespecified subgroup (P-interaction>0.10 throughout) and all eleven sensitivity scenarios.

**Conclusions:**

BCIS is independently associated with pathological response and survival under neoadjuvant PD-1-based immunochemotherapy and adds discrimination beyond tumor-centered biomarkers and clinical staging. Because every component comes from routine pretreatment workup at no extra cost, BCIS could feasibly inform prehabilitation, toxicity surveillance, and shared decision-making.

## Introduction

Gastric cancer is the fifth most common cancer worldwide, but its burden falls heaviest on East Asia, and China alone accounts for nearly half of new cases each year (1). Most Chinese patients still come to attention at a locally advanced stage. For these patients, current Chinese guidelines recommend neoadjuvant systemic therapy followed by radical gastrectomy with D2 lymphadenectomy (2, 3). Over the past decade the chemotherapy backbone has shifted away from older epirubicin-based triplet schedules toward fluoropyrimidine-platinum doublets. Among these doublets, SOX (S-1 plus oxaliplatin) and XELOX (capecitabine plus oxaliplatin) are the two most widely used at our centers. Each step in this evolution has produced modest but consistent gains in pathological response and long-term survival (4).

Adding programmed cell death-1 (PD-1) or programmed death-ligand 1 (PD-L1) inhibitors to the perioperative chemotherapy backbone has been the major change of recent years. CheckMate 649 first demonstrated overall survival benefit in the metastatic setting; perioperative trials including MATTERHORN, KEYNOTE-585, and DANTE then reported absolute pCR gains of roughly 8–16% over chemotherapy alone, together with higher R0 resection rates and early survival signals (5–7). Yet response varies widely between patients. Tumor-side biomarkers such as PD-L1 CPS, mismatch repair (MMR) status, and Epstein–Barr virus (EBV) status help to a degree but explain only part of the variability we see in clinic. By contrast, the host environment in which immune checkpoint inhibitors (ICIs) operate, particularly body composition, nutritional reserve, and systemic inflammation, has been examined far less in the curative-intent neoadjuvant context.

A number of individual host markers have already been linked to ICI outcomes across cancer types. CT-derived skeletal muscle index (SMI) and skeletal muscle density (SMD) at the L3 vertebral level have become routine radiologic surrogates for cachexia and myosteatosis, two states that may suppress T-cell metabolism and dampen treatment tolerance (8–10). On the laboratory side, the prognostic nutritional index (PNI), neutrophil-to-lymphocyte ratio (NLR), C-reactive protein-to-albumin ratio (CAR), and systemic immune-inflammation index (SII) have all been associated with ICI response in several solid tumors including gastric cancer (11–13). Most of this work has tested one or two markers at a time. Integrated tools that combine muscle quantity, muscle quality, visceral adiposity, nutritional reserve, inflammation, and anemia into one clinically interpretable score remain uncommon in gastric cancer (14–15).

In addition, a clinically pertinent counter-question is whether host-state burden modifies irAE risk. Several palliative ICI series have suggested that pre-existing inflammation may amplify irAE incidence, while others have not confirmed this association in perioperative cohorts (16, 17). Resolving whether an integrated host-state signature simultaneously predicts response and toxicity, selectively predicts response without affecting toxicity, or has no independent role at all, is of substantial clinical relevance because patients in this disease setting routinely present with concurrent cachexia, anemia, and inflammation.

Against this background, we designed a multicenter retrospective study in 720 patients with locally advanced gastric or EGJ adenocarcinoma treated with neoadjuvant PD-1 inhibitor plus chemotherapy. The Body Composition and Immunonutritional Signature (BCIS) was built as an additive integer score combining ten prespecified adverse host features. We then asked whether BCIS predicts pCR, MPR, ORR, and DCR; whether the relationship between continuous host-state variables and pCR is linear or nonlinear; whether BCIS strata differ in any-grade and Grade ≥3 immune-related adverse events (irAEs); whether BCIS independently predicts PFS and OS in Cox models; and whether adding BCIS to a clinical staging baseline produces meaningful gains in discrimination and clinical utility, tested by DeLong analysis and decision-curve analysis.

## Materials and methods

### Study design and patient population

Four tertiary hospitals in Hebei Province contributed cases. The Fourth Hospital of Hebei Medical University (HMU4H) was the lead center; the other three were Shijiazhuang People’s Hospital, Baoding Central Hospital, and Hengshui People’s Hospital. We screened all consecutive patients seen at any of these hospitals from January 2019 through December 2023 with a histologic diagnosis of gastric or EGJ adenocarcinoma. Each center’s institutional review board approved the protocol. Because the analysis was retrospective and used only de-identified records, written informed consent from the participants was not required to participate in this study in accordance with the national legislation and the institutional requirements. The study conformed to the Declaration of Helsinki. Of 1,156 patients screened, 436 (37.7%) were excluded for the reasons enumerated in **Supplementary Table 1**, leaving 720 patients for the analytic cohort. Compared with included patients, excluded patients were slightly older (median age 63 versus 61 years), had a higher proportion of clinical stage IVA disease (42.9% versus 36.5%, P = 0.026) and ECOG PS ≥1 (58.3% versus 51.8%, P = 0.029), but did not differ in sex distribution or EGJ proportion.

Eligibility required several conditions. Disease had to be at clinical stage cT2–4bNanyM0 under the 8th edition AJCC/UICC TNM, staged on contrast-enhanced CT and/or endoscopic ultrasound. The neoadjuvant regimen had to be a PD-1 inhibitor combined with SOX or XELOX, with at least two cycles delivered before surgery. Each patient then underwent radical gastrectomy with D2 lymphadenectomy. We also required an interpretable pretreatment abdominal CT covering the L3 vertebra, a complete pretreatment laboratory panel drawn within seven days of the first cycle, a documented response assessment, and adequate follow-up. Patients were excluded for any of the following: prior systemic anti-tumor therapy; active autoimmune disease on systemic corticosteroids; organ-transplant history; hereditary lipid disorder; M1 disease confirmed at any time point; another concurrent active malignancy; or missing key body-composition or pathology data. Of 1,156 patients screened, 720 cleared all criteria. Cohort allocation followed enrollment center and time window. The training cohort drew from HMU4H 2019–2021 (n=334). The internal validation cohort drew from HMU4H 2022–2023 (n=145). The external validation cohort pooled the three remaining hospitals (n=241). Enrollment windows were non-overlapping across the three cohorts.

**Figure 1:**
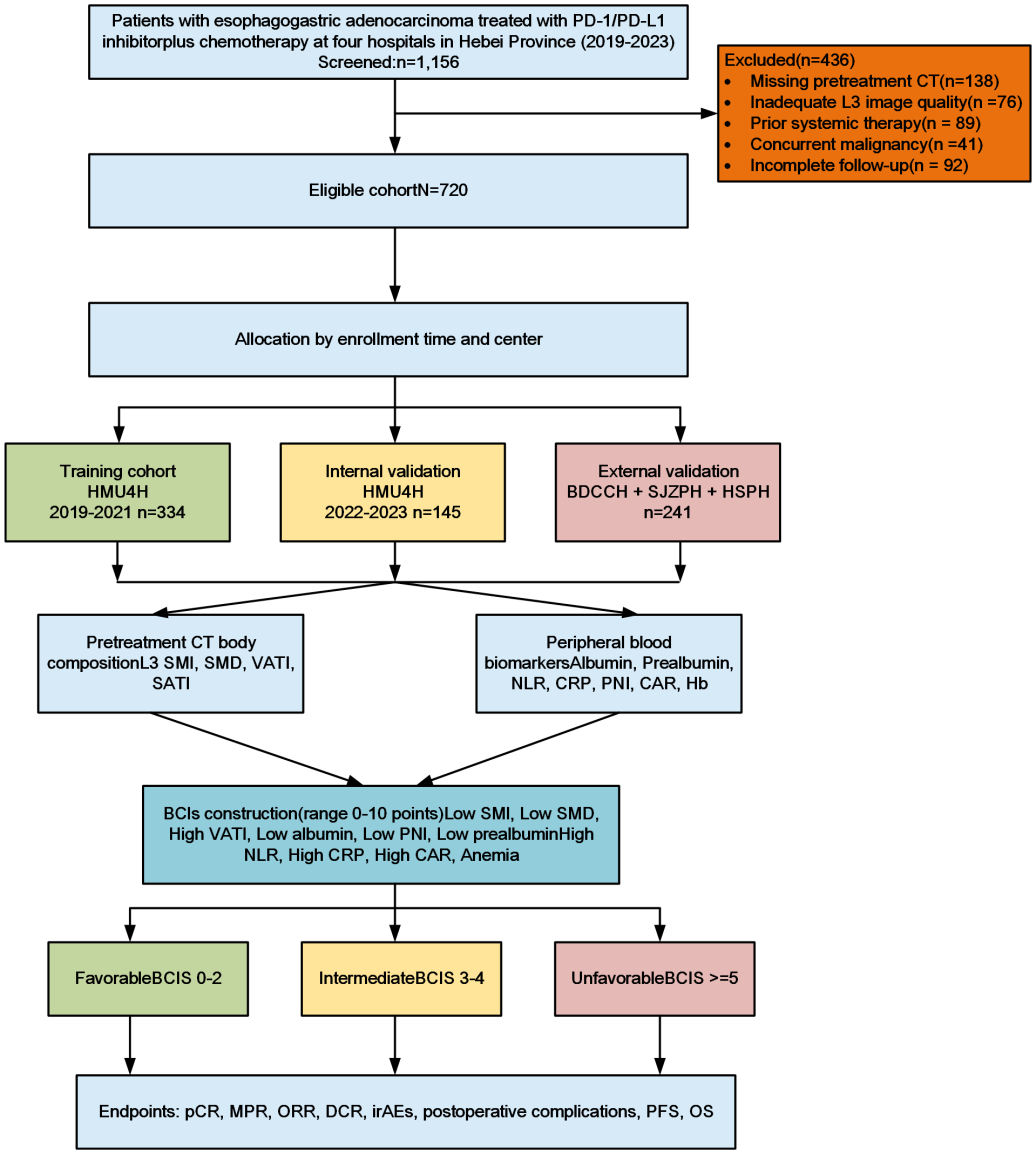
Patient flow and BCIS construction. The diagram shows screening and exclusion criteria, allocation to training, internal validation, and external validation cohorts, BCIS components, and study endpoints.

PD-1 inhibitors administered included sintilimab, camrelizumab, tislelizumab, toripalimab, pembrolizumab, and nivolumab. Pathological assessment was performed locally at each center, including tumor regression grading (Becker/Japanese system)(18), ypT and ypN staging, MMR status by immunohistochemistry for MLH1/MSH2/MSH6/PMS2, PD-L1 CPS testing on the Dako 22C3 platform, and EBV status by EBV-encoded RNA *in situ* hybridization. Discordant or borderline cases were reviewed centrally at the lead center. The flow of patient screening, allocation, and BCIS construction is summarized in **Figure 1**.

### BCIS construction and component assessment

Pretreatment CT body composition was quantified at the L3 vertebral level on the abdominal CT scan obtained closest to treatment initiation, by two radiologists blinded to clinical outcome using Sliceomatic software with predefined Hounsfield-unit thresholds (19). Skeletal muscle index (SMI), skeletal muscle density (SMD), visceral adipose tissue index (VATI), and subcutaneous adipose tissue index (SATI) were derived. Sex-specific thresholds based on prior Asian gastric cancer literature were applied: low SMI ≤40.8 cm²/m² (men) or ≤34.9 cm²/m² (women); low SMD ≤40.0 HU (men) or ≤35.0 HU (women); high VATI ≥60.0 cm²/m². Interobserver reproducibility was assessed in a randomly selected subset of 80 patients whose L3 slices were independently segmented by two board-certified abdominal radiologists (with 8 and 12 years of experience, respectively); both readers were blinded to patient identifiers, treatment outcomes, and all clinical or pathological data. Two-way mixed-effects intraclass correlation coefficients (ICCs) for absolute agreement were 0.972 (95% CI 0.964–0.978) for SMI, 0.948 (0.935–0.958) for SMD, 0.989 (0.986–0.991) for VATI, and 0.987 (0.984–0.990) for SATI, all comfortably exceeding the conventional 0.90 threshold for excellent reproducibility (**Supplementary Table 2**). Component cutoffs together with the principal literature sources and clinical interpretation are listed in **Supplementary Table 3**.

All laboratory measurements were obtained within seven days before treatment initiation. Composite indices were calculated as: PNI = albumin (g/L) + 5 × lymphocyte count (10^9^/L); NLR = neutrophil/lymphocyte ratio; PLR = platelet/lymphocyte ratio; LMR = lymphocyte/monocyte ratio; SII = neutrophil × platelet/lymphocyte; SIRI = neutrophil × monocyte/lymphocyte; CAR = CRP/albumin; HALP = (hemoglobin × albumin × lymphocyte)/platelet.

BCIS was constructed as an additive integer score (range 0–10) in which one point was assigned for each of the ten prespecified adverse pretreatment features: low SMI, low SMD, high VATI, albumin <38 g/L, PNI <47, prealbumin <180 mg/L, NLR >3, CRP >10 mg/L, CAR >0.25, and anemia (hemoglobin <120 g/L in men, <110 g/L in women). All component thresholds were prespecified from published literature (8, 11, 12, 14) and locked before validation analyses. Patients were assigned to a favorable (BCIS 0–2), intermediate (BCIS 3–4), or unfavorable (BCIS ≥5) group, with cutoffs corresponding approximately to the lower, middle, and upper tertiles of the BCIS distribution in independent gastric cancer cohorts.

### Outcome definitions

The primary efficacy endpoint was MPR (TRG 0–1, residual viable tumor ≤10%) as assessed by a board-certified gastrointestinal pathologist at each center. Secondary endpoints included pCR (TRG 0, no residual viable tumor), objective response rate (ORR) and disease control rate (DCR) per RECIST v1.1 (20), ypT/ypN downstaging rates, irAE incidence and severity per CTCAE v5.0, postoperative complications graded per the Clavien–Dindo classification, PFS (interval from treatment initiation to disease progression, recurrence, or death from any cause), and OS (interval from treatment initiation to death from any cause). Patients without an event were censored at the date of last follow-up.

### Statistical analysis

Continuous variables are reported as median (interquartile range, IQR) and categorical variables as n (%). Mann–Whitney U and χ²/Fisher’s exact tests were used for two-group comparisons; Kruskal–Wallis tests were used for three-group comparisons across BCIS strata. Univariable and multivariable logistic regression evaluated independent predictors of pCR and MPR, with results expressed as odds ratios (OR) and 95% CIs. Cochran–Armitage trend tests were used to evaluate monotonic trends across BCIS strata for binary outcomes.

Restricted cubic spline (RCS) analyses with four knots placed at the 5th, 35th, 65th, and 95th percentiles were performed within multivariable logistic regression to assess potential nonlinear relationships between continuous body-composition and immunonutritional variables (BMI, SMI, SMD, VATI, albumin, prealbumin, hemoglobin, CRP, NLR, PNI, CAR, BCIS total score) and pCR probability. P-overall reflected joint significance of spline basis terms; P-nonlinearity was derived from a likelihood-ratio test comparing the spline model to a linear-term model on the same covariate.

Kaplan–Meier curves were compared by log-rank test. Multivariable Cox proportional hazards regression with a small ridge penalty (penalizer=0.05) for numerical stability identified independent prognostic factors for PFS and OS, adjusting for age, sex, cT, cN, pCR (for survival models), PD-L1 CPS ≥5, MMR status, EGJ origin, and chemotherapy regimen. Prespecified subgroup analyses evaluated BCIS effect heterogeneity across age, sex, tumor location, cT/cN stage, PD-L1 CPS, MMR status, diabetes history, and chemotherapy regimen; P-interaction was derived from likelihood-ratio tests on multiplicative interaction terms.

To formally evaluate the predictive value of BCIS, two nested logistic regression models for MPR were constructed: (Model 1) clinical baseline (age, ECOG, cT, cN, EGJ, PD-L1 CPS, MMR, regimen); (Model 2) Model 1 plus BCIS total score. Discrimination (AUC for MPR) was estimated with 1000-bootstrap resampling for 95% CIs, and pairwise discrimination was compared using the DeLong test. Calibration was assessed with the Hosmer–Lemeshow test and bootstrap calibration plots; clinical utility was assessed with decision-curve analysis (DCA). Eleven sensitivity analyses were performed: excluding patients with diabetes, hypertension, age ≥70 years, ECOG PS ≥2, surgery-only cohort, R0 resection-only cohort, lead-center cohort, external validation only, and BCIS re-derivation excluding the high VATI and anemia components individually.

Spearman rank correlation flagged any pair of continuous variables with |ρ| above 0.5. Variance inflation factors (VIFs) supplemented this check; we did not co-enter a pair of collinear variables when one had a VIF above 5. Primary analyses ran in Python 3.11, using statsmodels, lifelines, scikit-learn, and patsy. We cross-validated key estimates in R 4.4.0 with the rms, pROC, survminer, and ggplot2 packages. P values below 0.05, two-sided, were taken as statistically significant. The primary multivariable models were additionally adjusted for the number of neoadjuvant cycles to account for treatment-exposure heterogeneity. To address residual confounding by baseline imbalance across BCIS groups, we performed inverse probability of treatment weighting (IPTW) using propensity scores estimated from a logistic model containing age, sex, ECOG ≥1, diabetes, hypertension, cT4, cN2/N3, EGJ origin, PD-L1 CPS ≥5, MMR status, and chemotherapy backbone, and report standardized mean differences (SMDs) before and after weighting in **Supplementary Table 4**. Each of the ten BCIS components was further evaluated separately by univariable and multivariable logistic regression for MPR, and a leave-one-component-out analysis was performed by rebuilding nine-component scores in turn (**Supplementary Table 5**). A regression-weighted alternative was constructed by LASSO-penalized logistic regression on the training cohort and compared with the equal-weight BCIS in all three cohorts (**Supplementary Table 6**). Six redundancy-removed BCIS variants excluding one variable from each highly correlated pair (low albumin versus low PNI; high CRP versus high CAR) were also tested (**Supplementary Table 7**). For survival analyses, primary Cox models were specified without pCR adjustment (as pCR is a post-treatment variable on the BCIS-survival causal pathway), with pCR-adjusted models retained as a secondary specification; the proportional-hazards assumption was tested using scaled Schoenfeld residuals with the rank time transformation. Predictive performance was further quantified by continuous net reclassification improvement (NRI), integrated discrimination improvement (IDI), calibration-in-the-large, and calibration slope; cohort-stratified decision-curve analysis was tabulated at four prespecified threshold probabilities (0.20, 0.30, 0.40, 0.50), chosen to span the clinically meaningful action range from prehabilitation referral (lower threshold) to consideration of regimen alternatives (higher threshold). Center-specific external validation was reported separately for each of the three external hospitals (**Supplementary Table 8**).

## Results

### Patient characteristics and BCIS distribution

Out of 1,156 screened patients, 720 entered the analysis: 334 in the training cohort, 145 in the internal validation cohort, and 241 in the external validation cohort. Median age at diagnosis was 61 years (IQR 56–68). Most were men (539, 74.9%). Siewert II/III EGJ tumors accounted for 179 cases (24.9%). Locally advanced features were the rule rather than the exception, with 343 patients (47.6%) at cT4 and 393 (54.6%) at cN2/cN3. PD-L1 CPS reached 5 or higher in 377 patients (52.4%), and 59 (8.2%) had dMMR/MSI-H tumors. SOX served as the chemotherapy backbone for 467 patients (64.9%) and XELOX for the remaining 253 (35.1%); the median number of preoperative cycles was four. R0 resection was eventually achieved in 610 patients (84.7%). **Table 1** lists the full baseline profile by BCIS stratum, summarized in **Table 1**.

**Table 1:** Baseline clinical and tumor characteristics of the study population stratified by BCIS group.

Characteristic	Overall (n=720)	Favorable BCIS (n=243)	Intermediate BCIS (n=303)	Unfavorable BCIS (n=174)	P value
Age, years, median (IQR)	61 (56–68)	60 (54–67)	61 (56–67)	64 (58–70)	<0.001
Male sex, n (%)	539 (74.9)	181 (74.5)	230 (75.9)	128 (73.6)	0.823
BMI, kg/m², median (IQR)	22.7 (20.5–25.3)	22.5 (20.6–24.7)	22.7 (20.4–25.5)	22.9 (20.5–25.7)	0.520
ECOG PS ≥1, n (%)	373 (51.8)	108 (44.4)	156 (51.5)	109 (62.6)	0.001
Current smoker, n (%)	143 (19.9)	42 (17.3)	62 (20.5)	39 (22.4)	0.396
Diabetes, n (%)	112 (15.6)	21 (8.6)	45 (14.9)	46 (26.4)	<0.001
Hypertension, n (%)	255 (35.4)	72 (29.6)	107 (35.3)	76 (43.7)	0.013
EGJ adenocarcinoma, n (%)	179 (24.9)	67 (27.6)	75 (24.8)	37 (21.3)	0.350
Clinical T4 (cT4a/cT4b), n (%)	343 (47.6)	107 (44.0)	138 (45.5)	98 (56.3)	0.025
Clinical N2/N3, n (%)	393 (54.6)	111 (45.7)	170 (56.1)	112 (64.4)	<0.001
Clinical stage IVA, n (%)	263 (36.5)	77 (31.7)	107 (35.3)	79 (45.4)	0.013
Diffuse Lauren type, n (%)	284 (39.4)	91 (37.4)	123 (40.6)	70 (40.2)	0.728
Poorly/undifferentiated, n (%)	578 (80.3)	187 (77.0)	246 (81.2)	145 (83.3)	0.234
PD-L1 CPS ≥5, n (%)	377 (52.4)	139 (57.2)	155 (51.2)	83 (47.7)	0.150
dMMR/MSI-H, n (%)	59 (8.2)	22 (9.1)	25 (8.3)	12 (6.9)	0.737
HER2-positive, n (%)	125 (17.4)	42 (17.3)	51 (16.8)	32 (18.4)	0.913
EBV-positive, n (%)	39 (5.4)	13 (5.3)	17 (5.6)	9 (5.2)	0.974
SOX backbone, n (%)	467 (64.9)	155 (63.8)	199 (65.7)	113 (64.9)	0.898
XELOX backbone, n (%)	253 (35.1)	88 (36.2)	104 (34.3)	61 (35.1)	0.898
Neoadjuvant cycles, median (IQR)	4 (4–5)	4 (4–5)	4 (4–5)	4 (3–5)	0.069

Continuous variables compared by Kruskal–Wallis test, categorical variables by χ² or Fisher’s exact test as appropriate. BCIS, body composition and immunonutritional signature; ECOG, Eastern Cooperative Oncology Group; EGJ, esophagogastric junction; PD-L1, programmed death-ligand 1; CPS, combined positive score; dMMR, deficient mismatch repair; MSI-H, microsatellite instability high; HER2, human epidermal growth factor receptor 2; EBV, Epstein–Barr virus; SOX, S-1 plus oxaliplatin; XELOX, capecitabine plus oxaliplatin.

Across the full cohort, 188 (26.1%) patients had low SMI, 342 (47.5%) low SMD, 91 (12.6%) high VATI, 286 (39.7%) low albumin, 368 (51.1%) low PNI, 274 (38.1%) low prealbumin, 328 (45.6%) high NLR, 162 (22.5%) high CRP, 166 (23.1%) high CAR, and 172 (23.9%) anemia. Median BCIS total score was 3 (IQR 2–4). Applying prespecified cutoffs, 243 (33.8%) patients were classified as favorable, 303 (42.1%) as intermediate, and 174 (24.2%) as unfavorable. BCIS group distribution was consistent across the training, internal validation, and external validation cohorts (all between-cohort P≥0.42), supporting the comparability of cohorts. As expected, unfavorable BCIS patients were older, more frequently had ECOG PS ≥1, diabetes, hypertension, cT4 disease, and cN2/cN3 disease, but did not differ from favorable patients in PD-L1 CPS, MMR status, HER2 status, EBV status, EGJ origin, or chemotherapy regimen (**Table 1**). BCIS components and composite indices according to MPR status are summarized in **Table 2**.

**Table 2:** BCIS components and composite immunometabolic indices stratified by MPR status.

Variable	Overall (n=720)	MPR (n=314)	Non-MPR (n=406)	P value
SMI, cm²/m², median (IQR)	44.1 (38.2–49.5)	44.8 (40.5–50.5)	43.6 (37.2–48.9)	0.007
SMD, HU, median (IQR)	39.2 (33.8–44.2)	40.2 (34.9–44.8)	38.9 (33.1–44.0)	0.073
VATI, cm²/m², median (IQR)	34.7 (21.5–49.6)	33.7 (20.7–47.5)	35.8 (22.3–51.0)	0.292
Albumin, g/L, median (IQR)	39.0 (36.1–41.6)	39.9 (37.2–42.3)	38.3 (35.8–41.1)	<0.001
Prealbumin, mg/L, median (IQR)	194 (162–229)	200 (168–231)	190 (158–228)	0.060
Hemoglobin, g/L, median (IQR)	130 (118–143)	133 (122–147)	127 (115–140)	<0.001
CRP, mg/L, median (IQR)	3.9 (1.7–9.2)	3.4 (1.6–6.3)	4.6 (1.8–11.8)	<0.001
NLR, median (IQR)	2.9 (1.9–4.0)	2.7 (1.8–3.8)	3.0 (1.9–4.1)	0.115
PNI, median (IQR)	46.8 (43.8–50.4)	47.8 (44.4–50.9)	46.0 (43.5–49.8)	<0.001
CAR, median (IQR)	0.10 (0.04–0.24)	0.08 (0.04–0.16)	0.12 (0.05–0.30)	<0.001
HALP, median (IQR)	32.5 (23.2–44.5)	34.5 (23.9–47.7)	31.6 (23.0–42.1)	0.030
Low SMI, n (%)	188 (26.1)	60 (19.1)	128 (31.5)	<0.001
Low SMD, n (%)	342 (47.5)	135 (43.0)	207 (51.0)	0.040
High VATI, n (%)	91 (12.6)	39 (12.4)	52 (12.8)	0.966
Low albumin (<38 g/L), n (%)	286 (39.7)	96 (30.6)	190 (46.8)	<0.001
Low PNI (<47), n (%)	368 (51.1)	142 (45.2)	226 (55.7)	0.007
Low prealbumin (<180 mg/L), n (%)	274 (38.1)	104 (33.1)	170 (41.9)	0.020
High NLR (>3), n (%)	328 (45.6)	124 (39.5)	204 (50.2)	0.005
High CRP (>10 mg/L), n (%)	162 (22.5)	45 (14.3)	117 (28.8)	<0.001
High CAR (>0.25), n (%)	166 (23.1)	48 (15.3)	118 (29.1)	<0.001
Anemia, n (%)	172 (23.9)	50 (15.9)	122 (30.0)	<0.001
BCIS total score, median (IQR)	3 (2–4)	3 (2–4)	4 (3–5)	<0.001
BCIS Favorable (0–2), n (%)	243 (33.8)	156 (49.7)	87 (21.4)	<0.001
BCIS Intermediate (3–4), n (%)	303 (42.1)	120 (38.2)	183 (45.1)	<0.001
BCIS Unfavorable (≥5), n (%)	174 (24.2)	38 (12.1)	136 (33.5)	<0.001

Continuous variables compared by Mann–Whitney U test, categorical variables by χ² test. SMI, skeletal muscle index; SMD, skeletal muscle density; VATI, visceral adipose tissue index; CRP, C-reactive protein; NLR, neutrophil-to-lymphocyte ratio; PNI, prognostic nutritional index; CAR, CRP-to-albumin ratio; HALP, hemoglobin-albumin-lymphocyte-platelet score; MPR, major pathological response.

### BCIS and pathological response

**Figure 2:**
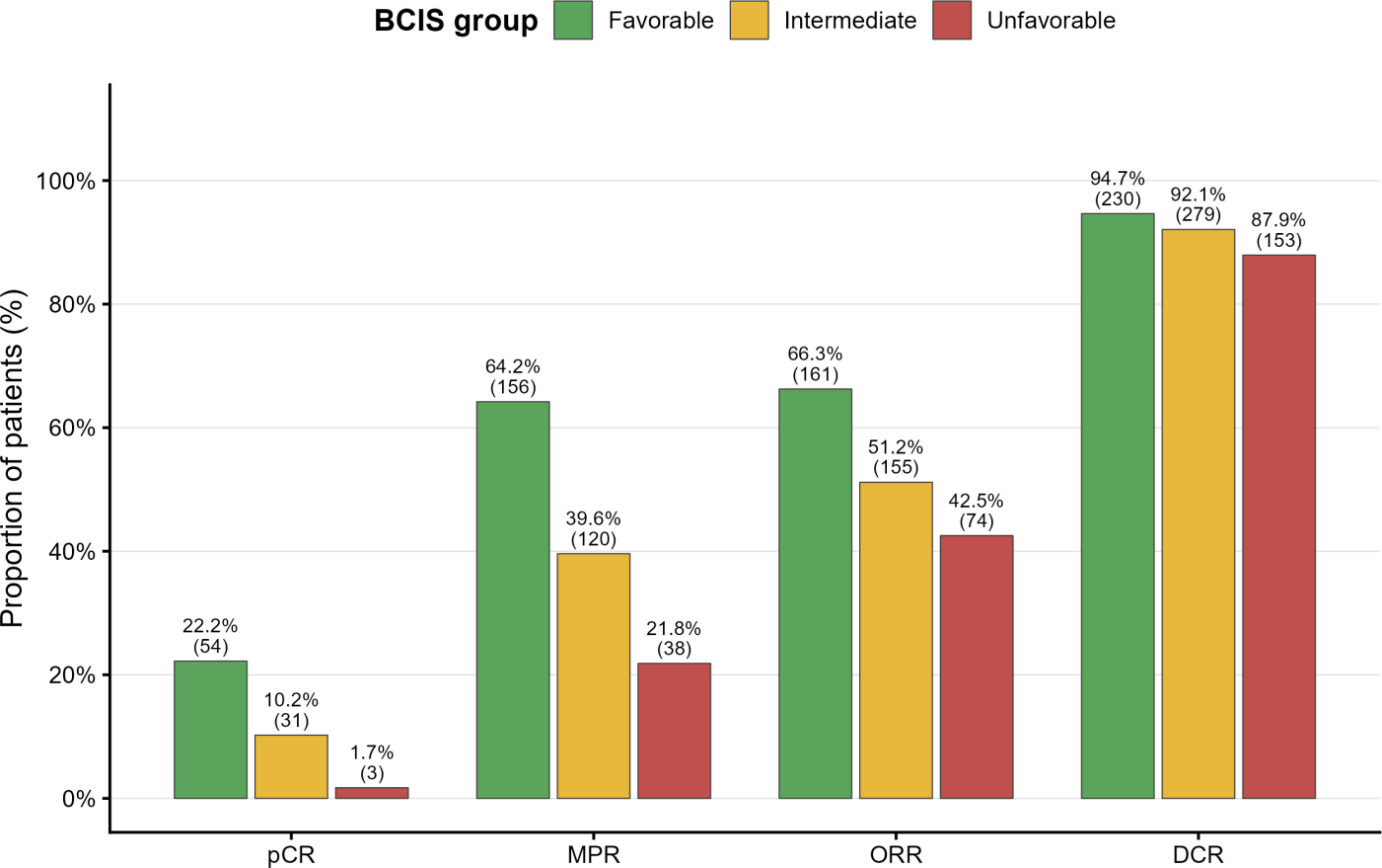
pCR, MPR, ORR, and DCR rates stratified by BCIS risk group. Bars show percentages within each group with absolute counts above each bar; χ² P<0.001 for pCR, MPR, and ORR; P = 0.045 for DCR.

The overall cohort pCR rate was 12.2% (88/720) and MPR rate was 43.6% (314/720). pCR rates declined progressively across BCIS groups (favorable 22.2% vs. intermediate 10.2% vs. unfavorable 1.7%; χ² P<0.001; Cochran–Armitage P-trend<0.001), as did MPR rates (64.2% vs. 39.6% vs. 21.8%; χ² P<0.001; P-trend<0.001), ORR (66.3% vs. 51.2% vs. 42.5%; P<0.001), and ypT0–2 downstaging (79.4% vs. 65.3% vs. 53.4%; P<0.001) (**Figure 2**; **Table 3**).

**Table 3:** Pathological and radiographic response, and downstaging across BCIS risk groups.

Outcome	Favorable (n=243)	Intermediate (n=303)	Unfavorable (n=174)	P value
pCR (TRG 0), n (%)	54 (22.2)	31 (10.2)	3 (1.7)	<0.001
MPR (TRG 0–1), n (%)	156 (64.2)	120 (39.6)	38 (21.8)	<0.001
ORR (CR+PR per RECIST 1.1), n (%)	161 (66.3)	155 (51.2)	74 (42.5)	<0.001
DCR (CR+PR+SD), n (%)	230 (94.7)	279 (92.1)	153 (87.9)	0.045
ypT0–2 downstaging, n (%)	193 (79.4)	198 (65.3)	93 (53.4)	<0.001
ypN0 downstaging, n (%)	176 (72.4)	174 (57.4)	82 (47.1)	<0.001
R0 resection, n (%)	206 (84.8)	262 (86.5)	142 (81.6)	0.365
Cochran–Armitage P-trend	—	—	—	<0.001 (pCR), <0.001 (MPR)

Three-group comparisons by χ² test; P-trend by Cochran–Armitage test for binary outcomes. pCR, pathological complete response; MPR, major pathological response; TRG, tumor regression grade; ORR, objective response rate; DCR, disease control rate.

In the multivariable logistic model adjusting for age, sex, ECOG performance status, cT/cN stage, EGJ origin, PD-L1 CPS, MMR status, and chemotherapy regimen, each one-point BCIS increase was independently associated with reduced odds of pCR (adjusted OR = 0.55, 95% CI 0.46–0.66, P<0.001) and MPR (adjusted OR = 0.61, 95% CI 0.55–0.68, P<0.001). When BCIS was modeled as a categorical exposure, the unfavorable group had markedly reduced odds of pCR compared with the favorable group (adjusted OR = 0.07, 95% CI 0.02–0.23, P<0.001) and of MPR (adjusted OR = 0.16, 95% CI 0.10–0.24, P<0.001). PD-L1 CPS ≥5 (adjusted OR for MPR = 1.60, 95% CI 1.15–2.22, P = 0.005) and dMMR/MSI-H status (adjusted OR = 2.77, 95% CI 1.51–5.10, P = 0.001) retained independent favorable associations with MPR, whereas cN2/cN3 disease (adjusted OR = 0.67, 95% CI 0.48–0.93, P = 0.016) was independently associated with reduced MPR odds (**Table 4**). Chemotherapy regimen and EGJ origin were not independent predictors of either endpoint.

**Table 4:** Multivariable logistic regression for pCR and MPR.

Variable	pCR adjusted OR (95% CI)	P value	MPR adjusted OR (95% CI)	P value
BCIS total score, per 1-point increase	0.55 (0.46–0.66)	<0.001	0.61 (0.55–0.68)	<0.001
BCIS Intermediate vs. Favorable	0.42 (0.26–0.69)	0.001	0.36 (0.25–0.51)	<0.001
BCIS Unfavorable vs. Favorable	0.07 (0.02–0.23)	<0.001	0.16 (0.10–0.24)	<0.001
Age, per year	1.01 (0.99–1.04)	0.353	1.00 (0.98–1.02)	0.904
Male sex	1.06 (0.61–1.83)	0.838	1.04 (0.74–1.46)	0.832
ECOG PS ≥1	0.71 (0.42–1.20)	0.205	0.73 (0.53–1.02)	0.062
Clinical T4	0.74 (0.43–1.27)	0.281	0.81 (0.59–1.12)	0.208
Clinical N2/N3	0.59 (0.34–1.01)	0.054	0.67 (0.48–0.93)	0.016
EGJ origin (Siewert II/III)	1.21 (0.69–2.13)	0.499	1.42 (0.98–2.05)	0.065
PD-L1 CPS ≥5	1.62 (0.95–2.76)	0.075	1.60 (1.15–2.22)	0.005
dMMR/MSI-H	3.21 (1.42–7.27)	0.005	2.77 (1.51–5.10)	0.001
XELOX vs. SOX backbone	0.96 (0.55–1.66)	0.876	0.93 (0.67–1.31)	0.685

Models were adjusted for age, sex, ECOG performance status, cT and cN stage, EGJ origin, PD-L1 CPS, MMR status, and chemotherapy regimen. BCIS was modeled both as a continuous variable (per 1-point increase) and as a 3-level categorical variable. OR, odds ratio; CI, confidence interval.

### Restricted cubic spline analyses

Spline modeling of the twelve continuous host-state variables produced a fairly clear picture. Two markers showed clear nonlinearity in their relationship with MPR: CRP (P-overall<0.001, P-nonlinearity=0.021) and the closely related CAR (P-overall<0.001, P-nonlinearity=0.053). For both, MPR probability fell steeply across the low-to-moderate range and then leveled off at higher values, a pattern that suggests saturation once systemic inflammation crosses a threshold. BMI (P-overall=0.412, P-nonlinearity=0.836), SMD (0.215, 0.184), VATI (0.288, 0.604), and prealbumin (0.228, 0.409) showed neither an overall nor a nonlinear signal. Four other variables—albumin, hemoglobin, PNI, and SMI—were significant in the overall test but flat on the nonlinearity test (all P-nonlinearity ≥0.18), so they entered later models in linear form. The BCIS total score itself was strictly linear (P-overall<0.001, P-nonlinearity=0.537), which justified its use as a continuous integer in multivariable models without further transformation (**Table 5**).

**Table 5:** Restricted cubic spline analyses of continuous variables and pCR/MPR probability.

Continuous variable	Knot positions (5th, 35th, 65th, 95th percentile)	P-overall	P-nonlinearity
Body mass index, kg/m²	17.9, 21.3, 23.7, 28.4	0.412	0.836
Skeletal muscle index, cm²/m²	32.5, 41.5, 46.5, 56.7	0.018	0.291
Skeletal muscle density, HU	24.5, 36.1, 41.5, 51.8	0.215	0.184
Visceral adipose tissue index, cm²/m²	7.1, 27.5, 42.5, 70.9	0.288	0.604
Albumin, g/L	31.4, 37.5, 40.5, 45.7	<0.001	0.412
Prealbumin, mg/L	118, 178, 215, 273	0.228	0.409
Hemoglobin, g/L	100, 124, 138, 158	<0.001	0.193
CRP, mg/L	0.5, 2.6, 6.6, 32.4	<0.001	0.021
NLR	1.4, 2.4, 3.4, 6.4	0.099	0.058
PNI	39.7, 45.4, 48.6, 53.5	0.023	0.462
CAR	0.013, 0.067, 0.169, 0.778	<0.001	0.053
BCIS total score	1, 3, 4, 7	<0.001	0.537

Spline models used four knots placed at the 5th, 35th, 65th, and 95th percentiles. P-overall reflects the joint significance of spline basis terms in a multivariable model adjusted for age, cT, cN, PD-L1 CPS, and MMR status. P-nonlinearity reflects the likelihood-ratio test comparing the spline model with a linear-term model on the same covariate.

### BCIS and immune-related adverse events

Any-grade irAEs occurred in 369 of 720 patients (51.2%), and 56 (7.8%) had Grade ≥3 events. The any-grade rate was somewhat higher in the unfavorable BCIS group than in the favorable group (59.8% vs. 48.6%, OR = 1.57, 95% CI 1.07–2.30, P = 0.036). Grade ≥3 irAEs, however, did not split cleanly by BCIS (favorable 6.6%, intermediate 7.6%, unfavorable 9.8%; P = 0.482). Treatment discontinuation related to irAE was numerically more frequent in the unfavorable group (6.9% vs. 3.7%) but the difference fell short of significance (P = 0.299). When we looked at organ-specific irAEs, including skin, hepatic, thyroid, and pulmonary events, no category reached statistical significance between BCIS strata (all P>0.18). Postoperative Clavien–Dindo grade ≥II complications followed a similar pattern: 25.9% in the unfavorable group versus 19.8% in the favorable group, OR = 1.42 (95% CI 0.89–2.27, P = 0.145), and again the trend did not reach significance (**Table 6**). These results indicate that BCIS-related host vulnerability is reflected primarily in mild-to-moderate immunologic toxicity and postoperative recovery rather than in life-threatening irAEs. Adjusted logistic regression models confirmed that each one-point BCIS increment was modestly but independently associated with any-grade irAEs after adjustment for age, sex, ECOG, diabetes, hypertension, and chemotherapy backbone (adjusted OR = 1.12, 95% CI 1.02–1.22, P = 0.017), whereas no independent association was detected with Grade ≥3 irAEs (adjusted OR = 1.08, 95% CI 0.92–1.28, P = 0.344) or with Clavien–Dindo grade ≥II postoperative complications (adjusted OR = 1.09, 95% CI 0.98–1.22, P = 0.113; **Supplementary Table 9**).

**Table 6:** Immune-related adverse events and postoperative complications across BCIS risk groups.

Adverse event	Favorable (n=243)	Intermediate (n=303)	Unfavorable (n=174)	OR (vs. favorable)	P value
Any-grade irAE, n (%)	118 (48.6)	147 (48.5)	104 (59.8)	1.57 (1.07–2.30)	0.036
Grade ≥3 irAE, n (%)	16 (6.6)	23 (7.6)	17 (9.8)	1.54 (0.75–3.16)	0.482
Treatment discontinuation due to irAE, n (%)	9 (3.7)	14 (4.6)	12 (6.9)	1.92 (0.79–4.66)	0.299
Skin/dermatologic irAE, n (%)	30 (12.3)	45 (14.9)	32 (18.4)	1.59 (0.93–2.74)	0.193
Hepatic irAE, n (%)	15 (6.2)	22 (7.3)	17 (9.8)	1.65 (0.80–3.40)	0.323
Endocrine irAE (thyroid), n (%)	38 (15.6)	55 (18.2)	30 (17.2)	1.13 (0.67–1.91)	0.880
Pneumonitis, n (%)	12 (4.9)	19 (6.3)	15 (8.6)	1.81 (0.83–3.95)	0.286
Postoperative CD ≥II complication, n (%)	48 (19.8)	56 (18.5)	45 (25.9)	1.42 (0.89–2.27)	0.145

Three-group comparisons by χ² or Fisher’s exact test; ORs are unfavorable vs. favorable. irAE, immune-related adverse event; CD, Clavien–Dindo classification.

### Survival analysis

**Figure 3:**
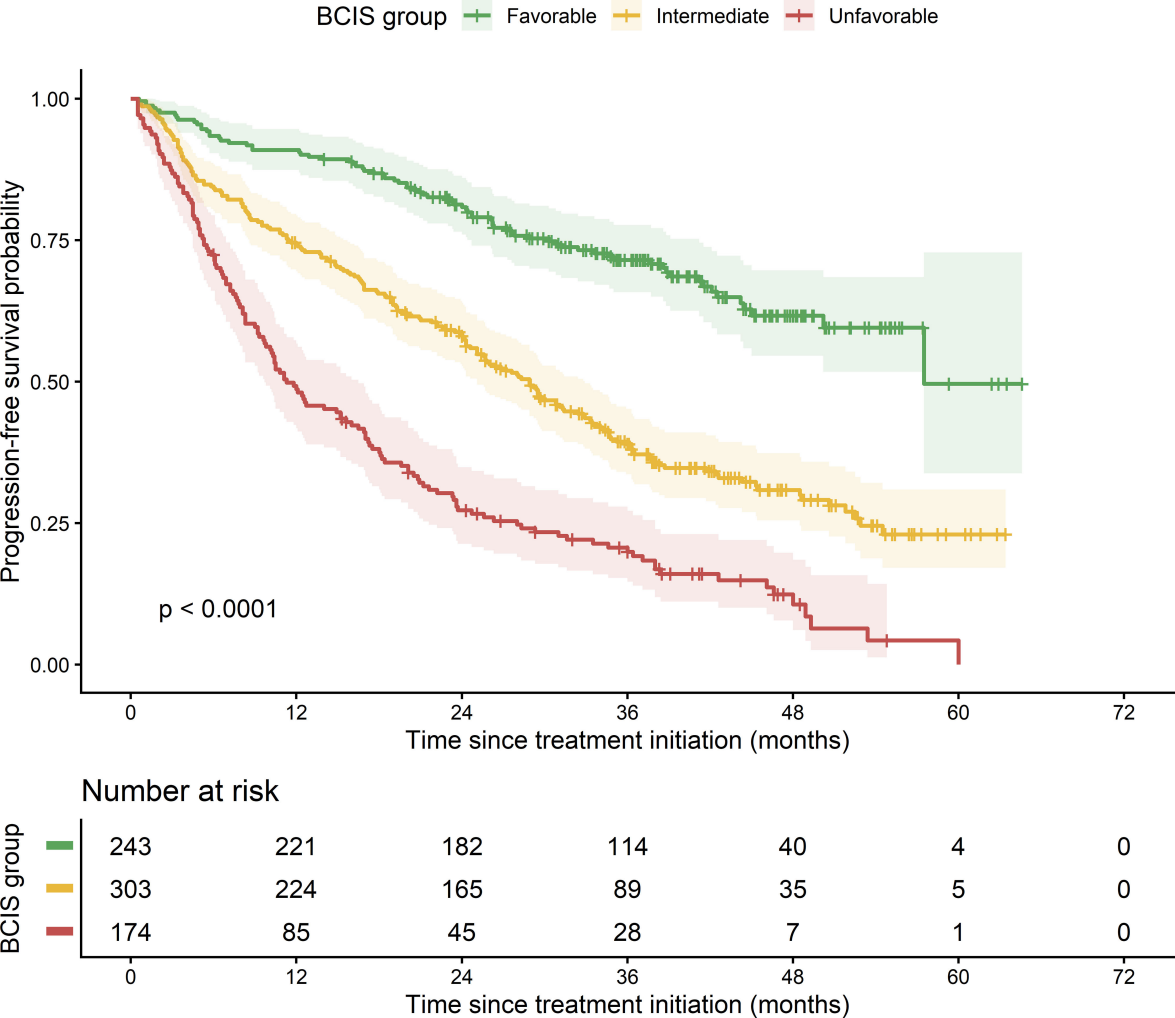
Kaplan–Meier curves for progression-free survival stratified by BCIS risk group. Number at risk is shown below each panel. Log-rank P<0.001.

**Figure 4:**
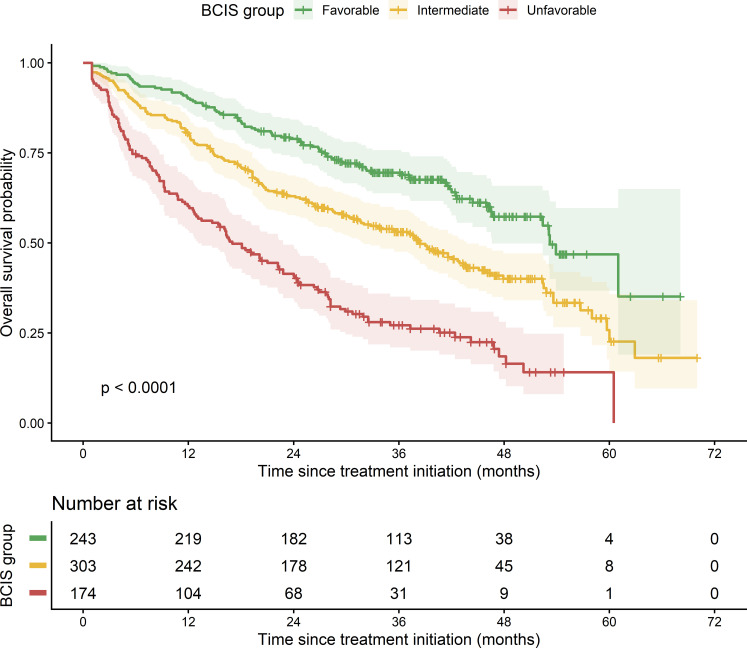
Kaplan–Meier curves for overall survival stratified by BCIS risk group. Number at risk is shown below each panel. Log-rank P<0.001.

At a median follow-up of 42.5 months (IQR 34.6–50.6), PFS events occurred in 425 (59.0%) and OS events in 390 (54.2%) patients. Median PFS was 35.0 (IQR 23.8–43.7), 25.6 (11.5–37.8), and 11.2 (5.3–24.6) months across favorable, intermediate, and unfavorable BCIS groups, respectively, and median OS was 34.3 (24.0–43.7), 29.6 (14.6–43.1), and 16.6 (5.7–30.6) months. Kaplan–Meier curves separated by BCIS group with log-rank P<0.001 for both PFS and OS (**Figures 3**, **4**). In multivariable Cox regression specified without pCR adjustment (so as not to over-adjust for a post-treatment variable on the BCIS–survival causal pathway) and adjusting for age, sex, cT, cN, PD-L1 CPS, MMR status, EGJ origin, and chemotherapy regimen, each one-point BCIS increase was independently associated with worse PFS (adjusted HR = 1.47, 95% CI 1.39–1.56, P<0.001) and worse OS (adjusted HR = 1.33, 95% CI 1.26–1.42, P<0.001). Estimates from the pCR-adjusted secondary specification were similar (PFS HR = 1.46, OS HR = 1.31), confirming that pCR mediates only a small portion of the BCIS prognostic signal (**Supplementary Table 10**). Proportional-hazards (PH) assumption testing using scaled Schoenfeld residuals showed the PH assumption was satisfied for PFS (P = 0.406) but mildly violated for OS (P = 0.015), which we interpret as reflecting the prolonged follow-up tail; time-stratified Cox analysis at 24 months yielded directionally consistent estimates (HR = 1.31 in months 0–24; HR = 1.42 thereafter). Categorical BCIS analysis showed substantially elevated PFS hazard for the unfavorable group (adjusted HR = 5.44, 95% CI 4.13–7.16) and OS hazard (adjusted HR = 3.41, 95% CI 2.60–4.47) versus favorable (**Table 7**). Achievement of pCR was independently protective for both PFS (adjusted HR = 0.31, 95% CI 0.22–0.43, P<0.001) and OS (adjusted HR = 0.46, 95% CI 0.32–0.65, P<0.001).

**Table 7:** Multivariable Cox regression for PFS and OS.

Variable	PFS HR (95% CI)	P value	OS HR (95% CI)	P value
BCIS total score, per 1-point increase	1.53 (1.44–1.63)	<0.001	1.38 (1.29–1.47)	<0.001
BCIS Intermediate vs. Favorable	2.39 (1.85–3.09)	<0.001	1.90 (1.47–2.45)	<0.001
BCIS Unfavorable vs. Favorable	5.44 (4.13–7.16)	<0.001	3.41 (2.60–4.47)	<0.001
Age, per year	1.01 (1.00–1.02)	0.258	0.99 (0.98–1.00)	0.216
Male sex	1.04 (0.83–1.30)	0.732	1.06 (0.84–1.34)	0.643
ECOG PS ≥1	1.07 (0.88–1.29)	0.522	1.05 (0.86–1.29)	0.630
Clinical T4	1.11 (0.92–1.35)	0.289	1.28 (1.05–1.56)	0.015
Clinical N2/N3	1.16 (0.95–1.41)	0.135	1.02 (0.83–1.26)	0.822
PD-L1 CPS ≥5	1.00 (0.82–1.22)	0.981	1.13 (0.92–1.39)	0.240
dMMR/MSI-H	0.75 (0.51–1.10)	0.144	0.89 (0.61–1.30)	0.547
XELOX vs. SOX backbone	1.05 (0.86–1.28)	0.643	0.97 (0.78–1.20)	0.760
EGJ origin	1.00 (0.80–1.25)	0.967	0.87 (0.68–1.10)	0.229
pCR achieved	0.31 (0.22–0.43)	<0.001	0.46 (0.32–0.65)	<0.001

Cox proportional hazards models with a small ridge penalty (penalizer=0.05). Both continuous and categorical BCIS specifications are presented; PFS models additionally adjust for pCR (binary) and OS models adjust for pCR. PFS, progression-free survival; OS, overall survival; HR, hazard ratio.

### Subgroup and Interaction analyses

Subgroup analyses across nine prespecified strata showed directionally consistent and individually significant adjusted ORs for the BCIS–MPR association in every subgroup that retained ≥50 events (**Table 8**). Adjusted ORs per BCIS point ranged from 0.55 (95% CI 0.47–0.65) in PD-L1 CPS ≥5 patients to 0.68 (95% CI 0.57–0.81) in patients receiving XELOX. Effects were significant in both sexes, both age strata, both EGJ and gastric subgroups, and in patients with and without diabetes. P-interaction values exceeded 0.10 for all evaluable strata, indicating no clinically meaningful effect modification (**Supplementary Tables 11** and **S12**; **Table 8**).

**Table 8:** Subgroup analyses of the multivariable BCIS–MPR association with P-interaction values.

Subgroup	n	Adjusted OR (95% CI) per BCIS point	P value	P-interaction
Sex				
Male	539	0.62 (0.55–0.70)	<0.001	0.576
Female	181	0.59 (0.46–0.74)	<0.001	
Age				
<65 years	451	0.58 (0.50–0.66)	<0.001	0.128
≥65 years	269	0.67 (0.55–0.80)	<0.001	
Tumor location				
Gastric (non-EGJ)	541	0.60 (0.53–0.68)	<0.001	0.342
EGJ adenocarcinoma	179	0.64 (0.52–0.78)	<0.001	
Clinical N stage				
cN0/cN1	327	0.58 (0.49–0.68)	<0.001	0.336
cN2/cN3	393	0.64 (0.55–0.74)	<0.001	
PD-L1 CPS				
CPS <5	343	0.68 (0.58–0.79)	<0.001	0.106
CPS ≥5	377	0.55 (0.47–0.65)	<0.001	
MMR status				
pMMR/MSS	661	0.60 (0.54–0.67)	<0.001	0.451
dMMR/MSI-H	59	0.66 (0.43–1.00)	0.050	
Chemotherapy backbone				
SOX	467	0.58 (0.50–0.67)	<0.001	0.124
XELOX	253	0.68 (0.57–0.81)	<0.001	
Diabetes				
No diabetes	608	0.62 (0.56–0.70)	<0.001	0.655
Diabetes	112	0.55 (0.40–0.76)	<0.001	

Each subgroup was analyzed by an independent multivariable logistic regression adjusted for the same covariates as the primary analysis (excluding the stratifying variable). P-interaction values were derived from likelihood-ratio tests on multiplicative interaction terms in the full cohort.

### Sensitivity analyses

In 11 prespecified sensitivity analyses, the adjusted OR for the BCIS–MPR association remained directionally consistent and statistically significant (**Table 9**). Excluding patients with diabetes (n=608, adjusted OR = 0.62, P<0.001), excluding patients with hypertension (n=465, adjusted OR = 0.58, P<0.001), excluding elderly patients (≥70 years, n=592, adjusted OR = 0.60, P<0.001), and restricting to ECOG PS 0–1 patients (n=661, adjusted OR = 0.63, P<0.001) produced highly similar effect sizes. Re-derivation of BCIS excluding the high VATI component (adjusted OR = 0.62, P<0.001) or the anemia component (adjusted OR = 0.63, P<0.001) did not change the conclusion, indicating that no single component dominated the additive signal. The lead-center cohort (HMU4H, n=479) and the external validation cohort (n=241) yielded adjusted ORs of 0.62 and 0.58 respectively, supporting the geographic transportability of the BCIS–MPR association. Inverse probability of treatment weighting (IPTW) achieved successful balancing of all 11 baseline covariates (|SMD|<0.10 after weighting; **Supplementary Table 4**), and the IPTW-weighted multivariable association between BCIS and MPR was virtually identical to the primary estimate (adjusted OR = 0.61, 95% CI 0.56–0.66, P<0.001), confirming that the effect was not driven by residual baseline imbalance. Six redundancy-removed BCIS variants excluding one variable from each closely correlated pair (low albumin versus low PNI; high CRP versus high CAR) yielded adjusted ORs ranging from 0.53 to 0.60, none materially different from the primary 10-component OR (**Supplementary Table 7**). Leave-one-component-out analyses produced adjusted ORs of 0.56 to 0.64 and AUCs of 0.671 to 0.694 across all ten variants (**Supplementary Table 5**), and a LASSO-penalized regression-weighted alternative differed from the equal-weight specification by only 0.008 in overall AUC and performed slightly worse in external validation (**Supplementary Table 6**), supporting retention of the equal-weight construction as the primary specification.

**Table 9:** Sensitivity analyses for the multivariable BCIS–MPR association.

Sensitivity scenario	n	Adjusted OR for MPR per BCIS point (95% CI)	P value
Primary analysis (full cohort)	720	0.61 (0.55–0.68)	<0.001
Excluding patients with diabetes	608	0.62 (0.56–0.70)	<0.001
Excluding patients with hypertension	465	0.58 (0.50–0.66)	<0.001
Excluding patients aged ≥70 years	592	0.60 (0.54–0.68)	<0.001
Excluding patients with ECOG PS ≥2	661	0.63 (0.56–0.71)	<0.001
Surgery-performed cohort only	683	0.60 (0.54–0.67)	<0.001
R0 resection cohort only	610	0.60 (0.53–0.67)	<0.001
Lead-center cohort (HMU4H, Train+Internal)	479	0.62 (0.54–0.71)	<0.001
External validation only	241	0.58 (0.47–0.71)	<0.001
Re-derivation excluding High VATI component	720	0.62 (0.56–0.70)	<0.001
Re-derivation excluding Anemia component	720	0.63 (0.57–0.71)	<0.001

Each row represents an independent multivariable logistic regression in the indicated subset, adjusted for the same covariates as the primary analysis where applicable. Re-derivation analyses excluded the indicated component when calculating BCIS but used the same prespecified cutoffs for the remaining components.

### Predictive model performance

To gauge what BCIS adds, we fit two nested logistic regressions for MPR. The clinical baseline (cT, cN, age, ECOG, EGJ origin, PD-L1 CPS, MMR, regimen) achieved an apparent training AUC of 0.628 (95% CI 0.568–0.679). Performance worsened on internal validation (AUC 0.530, 95% CI 0.436–0.624) and was modest externally (0.588, 95% CI 0.514–0.656). Pooled across all four cohorts, baseline AUC was 0.597 (0.553–0.642). Adding the BCIS total score lifted training AUC to 0.733 (DeLong P<0.001 vs. baseline), internal AUC to 0.652 (DeLong P = 0.073), external AUC to 0.704 (DeLong P = 0.014), and overall AUC to 0.710 (DeLong P<0.001). BCIS by itself, without any clinical covariates, already outperformed the full clinical baseline: external AUC 0.702 (95% CI 0.639–0.767), overall AUC 0.688 (95% CI 0.653–0.725), with overall DeLong P = 0.002 against the clinical baseline. Brier scores tracked the AUC trend, falling monotonically as BCIS was added (**Table 10**). Hosmer–Lemeshow tests showed acceptable calibration for all three model variants (all P>0.20). On decision-curve analysis, the combined model produced the largest net benefit over the 0.20–0.50 threshold range relevant to clinical decision-making, suggesting practical value beyond what staging alone provides. Reclassification metrics further supported the incremental contribution of BCIS: continuous net reclassification improvement (NRI) was +0.551 in training, +0.299 in internal validation, +0.572 in external validation, and +0.510 overall, with positive event and non-event components in every cohort; integrated discrimination improvement (IDI) was +0.124, +0.088, +0.116, and +0.115, respectively (all P<0.001; **Supplementary Table 13**). Calibration-in-the-large for the combined model improved from -0.202 (clinical baseline) to -0.127 in external validation, with calibration slope rising from 0.693 to 0.825; analogous gains were observed in internal validation (slope 0.298 → 0.737) and overall (slope 0.754 → 0.893). Center-specific external validation across the three external hospitals showed combined AUCs of 0.689 (Baoding), 0.696 (Hengshui), and 0.725 (Shijiazhuang) and tightly clustered center-specific BCIS-MPR adjusted ORs of 0.50, 0.54, and 0.55 (all P ≤ 0.010; **Supplementary Table 8**), supporting cross-center transportability of the BCIS signal. Cohort-stratified decision-curve net benefits at the prespecified thresholds of 0.20, 0.30, 0.40, and 0.50 are tabulated in **Supplementary Table 14**; at every threshold and in every cohort, the combined model produced higher net benefit than the clinical baseline.

**Table 10:** Predictive performance for MPR with DeLong tests against a clinical baseline.

Model	Cohort	AUC (95% CI)	Brier	DeLong P (vs. clinical baseline)
Clinical baseline (cT+cN+age+ECOG+EGJ+PD-L1+MMR+regimen)	Training	0.628 (0.568–0.679)	0.235	reference
Clinical baseline	Internal	0.530 (0.436–0.624)	0.251	reference
Clinical baseline	External	0.588 (0.514–0.656)	0.238	reference
Clinical baseline	Overall	0.597 (0.553–0.642)	0.240	reference
Clinical + BCIS total score	Training	0.733 (0.681–0.784)	0.206	<0.001
Clinical + BCIS total score	Internal	0.652 (0.548–0.735)	0.230	0.073
Clinical + BCIS total score	External	0.704 (0.643–0.771)	0.212	0.014
Clinical + BCIS total score	Overall	0.710 (0.670–0.747)	0.212	<0.001
BCIS total score alone	Training	0.696 (0.641–0.753)	0.218	0.012
BCIS total score alone	Internal	0.644 (0.558–0.734)	0.231	0.061
BCIS total score alone	External	0.702 (0.639–0.767)	0.214	0.018
BCIS total score alone	Overall	0.688 (0.653–0.725)	0.219	0.002

Models were trained on the training cohort and evaluated in each cohort. Cutoffs were fixed at the Youden index in the training cohort. AUC 95% CIs were derived from 1,000 bootstrap resamples. P values are DeLong tests against the clinical baseline within the same cohort.

### Collinearity assessment

Of the continuous body-composition and immunonutritional variables, four pairs reached |ρ|>0.5 on Spearman correlation: albumin with PNI (ρ=0.82, by definition), CRP with CAR (ρ=0.99, by definition), PNI with HALP (ρ=0.55), and PNI with the BCIS total score itself (ρ=–0.55). On the strength of these patterns we kept CRP and CAR out of the same model, and likewise for albumin and PNI; BCIS was always entered as the integrated score, not alongside its component features. VIFs in the primary MPR model came out uniformly low—BCIS total score 1.01, age 1.01, ECOG ≥1 1.02, cT4 1.01, cN2/cN3 1.02, PD-L1 CPS ≥5 1.05, dMMR/MSI-H 1.05, XELOX backbone 1.01, and EGJ origin 1.01—each well under the 5.0 threshold typically used to flag multicollinearity. These checks reinforce confidence in the primary multivariable inferences.

## Discussion

Three messages emerge from this multicenter cohort of 720 LAGC patients receiving neoadjuvant PD-1 inhibitor plus SOX or XELOX. The integrated 10-feature BCIS score behaved as an independent predictor of pCR, MPR, ORR, ypT/ypN downstaging, PFS, and OS, with the adjusted MPR odds ratio of 0.61 per point (95% CI 0.55–0.68, P<0.001) and the adjusted PFS hazard ratio of 1.53 per point (95% CI 1.44–1.63, P<0.001) anchoring its predictive weight. BCIS also added meaningful discrimination over a clinical baseline that already contained cT/cN stage, PD-L1 CPS, MMR status, EGJ origin, and chemotherapy regimen; the overall AUC for MPR climbed from 0.597 to 0.710 (DeLong P<0.001), and the external AUC moved from 0.588 to 0.704 (DeLong P = 0.014). Beyond that, the direction of the BCIS effect held up across all nine prespecified subgroups (P-interaction>0.10 throughout) and across 11 sensitivity scenarios, suggesting the signal is reasonably stable and geographically transportable within the Chinese LAGC population studied here.

A central conceptual question is whether BCIS captures additional prognostic information that PD-L1 CPS, MMR status, and conventional clinical staging do not. The DeLong tests answer this question affirmatively in the present cohort: PD-L1 CPS ≥5 and dMMR/MSI-H retained their established favorable associations with MPR (adjusted ORs 1.60 and 2.77, respectively) but together with full clinical staging contributed only modest discrimination (overall AUC 0.597). The addition of a single integer score derived from low-cost CT and blood inputs raised AUC by an absolute 11 percentage points overall and 12 percentage points externally. By comparison, the BCIS-alone overall AUC (0.688) approached the combined model AUC (0.710), suggesting that most of the incremental discriminatory information arises from host-state biology rather than from incremental tumor-centered information.

The biological plausibility of these findings rests on several converging mechanisms. Skeletal muscle is increasingly recognized as an immunologically active organ contributing to T-cell metabolism through release of branched-chain amino acids, glutamine, and myokines such as interleukin-15 and irisin, and reduction in muscle quantity or quality may impair the antitumor T-cell response that ICIs are designed to mobilize (9, 21). Visceral adiposity contributes to a pro-inflammatory adipokine milieu through tumor necrosis factor-α, interleukin-6, and leptin, and has been implicated in both impaired and amplified ICI activity in different settings (22). Hypoalbuminemia, low PNI, low prealbumin, anemia, elevated CRP, and elevated NLR collectively reflect a depleted nutritional reserve and a myeloid-dominant systemic inflammation state, both of which compromise effective antitumor immunity. The strength of an integrated signature, in contrast to single-marker approaches, is that no single component is required to capture the host milieu; rather, additive convergence across orthogonal biological domains delivers the predictive signal.

Our RCS analyses provide additional mechanistic insight. Two of twelve continuous variables, CRP and CAR, showed significant nonlinear associations with MPR, characterized by steep decline in response probability at low-to-moderate values and attenuation at high values, consistent with a ceiling effect of systemic inflammation on T-cell activity. The remaining ten continuous variables, including the BCIS total score itself, behaved linearly. This finding has direct relevance for predictive modeling: BCIS can be implemented as a simple integer score in clinical practice without spline transformation, while CRP-related markers may require nonlinear handling for fine-grained risk stratification.

The irAE findings are clinically reassuring with one important caveat. Although any-grade irAE incidence was modestly higher in the unfavorable BCIS group (59.8% vs. 48.6%, OR = 1.57), Grade ≥3 irAEs were not significantly different across strata (6.6–9.8%), and treatment discontinuation due to toxicity remained low (3.7–6.9%). These results indicate that LAGC patients with metabolically and nutritionally vulnerable host phenotypes can safely receive neoadjuvant PD-1 inhibitor combination therapy, although increased monitoring for mild-to-moderate immunologic toxicity may be warranted. Postoperative Clavien–Dindo grade ≥II complications were numerically but not statistically more common in the unfavorable group, consistent with the existing literature linking sarcopenia and hypoalbuminemia with delayed recovery after gastrectomy (8, 23).

The clinical implications of these findings are threefold. First, BCIS can be calculated at no marginal cost from data routinely captured in pretreatment workup and could be embedded in electronic medical record systems for automated risk stratification before neoadjuvant immunochemotherapy is initiated. Second, the unfavorable group, defined by BCIS ≥5, has substantially reduced odds of MPR (adjusted OR 0.16) and substantially elevated PFS hazard (adjusted HR 5.44) compared with the favorable group, defining a population that may benefit from intensified prehabilitation, including nutritional consultation, supervised resistance exercise, anemia correction, and treatment of correctable inflammation, ahead of immunochemotherapy. Whether such interventions improve pathological response and survival is the natural next prospective question. Third, the combined clinical-plus-BCIS model can serve as a baseline for future incorporation of additional biomarker domains, including dynamic on-treatment changes in body composition, microbiome composition, and circulating tumor-derived signals (24).

Several limitations are worth noting. As a retrospective study, ours is exposed to selection bias and to incomplete data capture, especially for irAE attribution, which is heavily dependent on chart documentation. All four participating hospitals sit in northern China; although BCIS held its performance in the external cohort, generalizing to non-Asian populations will require dedicated validation. Threshold values for the body-composition components were taken from previously published Asian gastric cancer cutoffs and may not be optimal for other ethnic groups or disease settings. We did not test FLOT chemotherapy, which is now the perioperative standard in many Western centers, simply because none of our participating institutions used FLOT during the enrollment period. Mechanistic underpinnings of BCIS—the tumor immune microenvironment, transcriptomic signatures, gut microbiome composition—were beyond the scope of the current dataset and will need separate translational work. While discrimination clearly improved with BCIS, the overall AUC of 0.710 still leaves predictive headroom; incorporation of pathomic and radiomic features could plausibly close some of that gap in future iterations (25). Several further caveats merit explicit acknowledgement. First, because all 720 patients received PD-1 inhibitor combined with chemotherapy, with no chemotherapy-only comparator arm, the present design cannot disentangle a general prognostic effect of BCIS from a PD-1-specific predictive effect; the results therefore support association with response and prognosis under neoadjuvant immunochemotherapy rather than a true immunotherapy-specific predictive role, and an external matched chemotherapy-only validation cohort is in protocol-design phase to address this question. Second, the proportional-hazards assumption was mildly violated for the BCIS coefficient in the OS Cox model (Schoenfeld P = 0.015), most likely reflecting the prolonged follow-up tail and the small number of OS events beyond 48 months; time-stratified sensitivity analysis at the 24-month landmark yielded directionally consistent estimates (HR = 1.31 early, HR = 1.42 late), so the qualitative conclusion is unchanged but readers should bear this nuance in mind. Third, the internal validation cohort AUC for the combined model (0.652) was lower than both training (0.733) and external validation (0.704); the most plausible contributors are the smaller sample size of the internal cohort (n=145), modest temporal variation in case-mix across 2022–2023, possible drift in supportive care and irAE recognition over the enrollment period, and random sampling variability at this sample size, with no evidence of systematic model failure since the external AUC (0.704) recovered close to training performance. These limitations notwithstanding, the consistency of the BCIS–outcome association across nine prespecified subgroups, eleven sensitivity scenarios, three external centers, and IPTW-balanced models supports the robustness of the principal findings.

## Conclusions

In 720 patients with locally advanced gastric or EGJ adenocarcinoma given neoadjuvant PD-1 inhibitor with SOX or XELOX, BCIS tracked closely with pathological response, with treatment safety, and with long-term survival. Its independent association persisted after full multivariable adjustment. Beyond a clinical baseline that already accounted for PD-L1 CPS, MMR status, and clinical staging, BCIS added a meaningful piece of discrimination, and the direction of effect held across every prespecified subgroup and every sensitivity scenario we examined. Host-state and tumor-immune information should therefore be combined when individualizing response prediction, not used in isolation. Prospective validation, mechanistic correlation, and trials of BCIS-guided prehabilitation are next on the agenda.

## Data Availability

The raw data supporting the conclusions of this article will be made available by the authors, without undue reservation.
