# Supplementary material for: An integrated body composition– immunonutritional signature for predicting immunotherapy response and prognosis in gastric cancer: a multicenter retrospective cohort study
Source: Front Oncol. 2026 Jul 15;16:1875482. doi: 10.3389/fonc.2026.1875482 (PMC13414903; doi:10.3389/fonc.2026.1875482)
Supplement: Supplementary file 1 [file DataSheet1.docx]

**Supplementary Figures and Tables**

An Integrated Body Composition–Immunonutritional Signature for Predicting Immunotherapy Response and Prognosis in Gastric Cancer

This file contains Supplementary Figures S1 to S2 and Supplementary Tables S1 to S14. Numbering follows order of first citation in the revised main text.

**
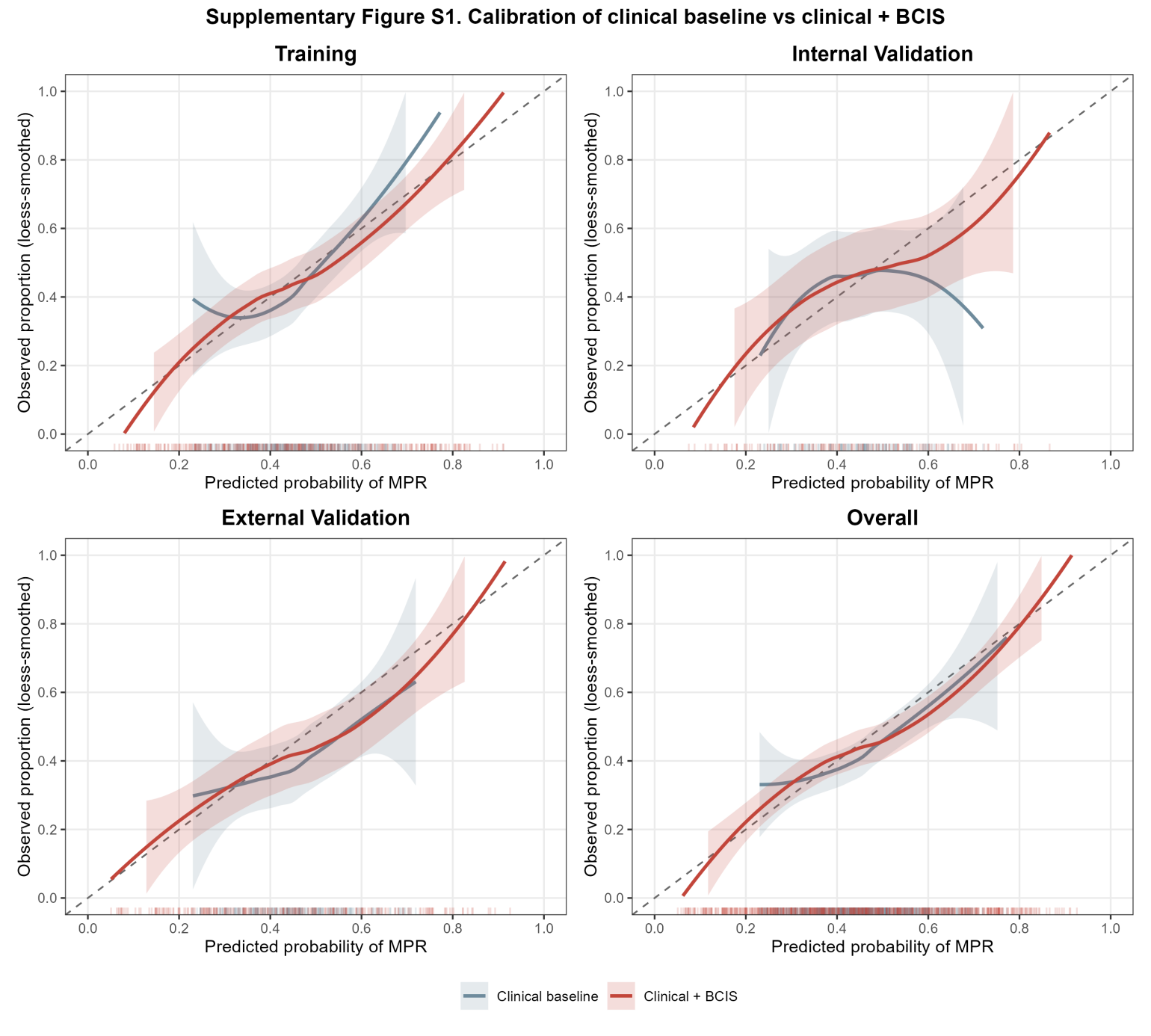
**

**Supplementary Figure S1.**

Calibration plots of the clinical baseline model and the clinical-plus-BCIS combined model for predicting MPR, shown in the training, internal validation, external validation, and overall cohorts. The loess-smoothed observed proportion of MPR is plotted against the predicted probability; the dashed 45° line indicates perfect calibration, and shaded bands are 95% confidence intervals. Rug marks at the bottom show predicted-probability density. The combined model (red) tracks the diagonal more closely than the clinical baseline (blue-gray) across all four cohorts.

**
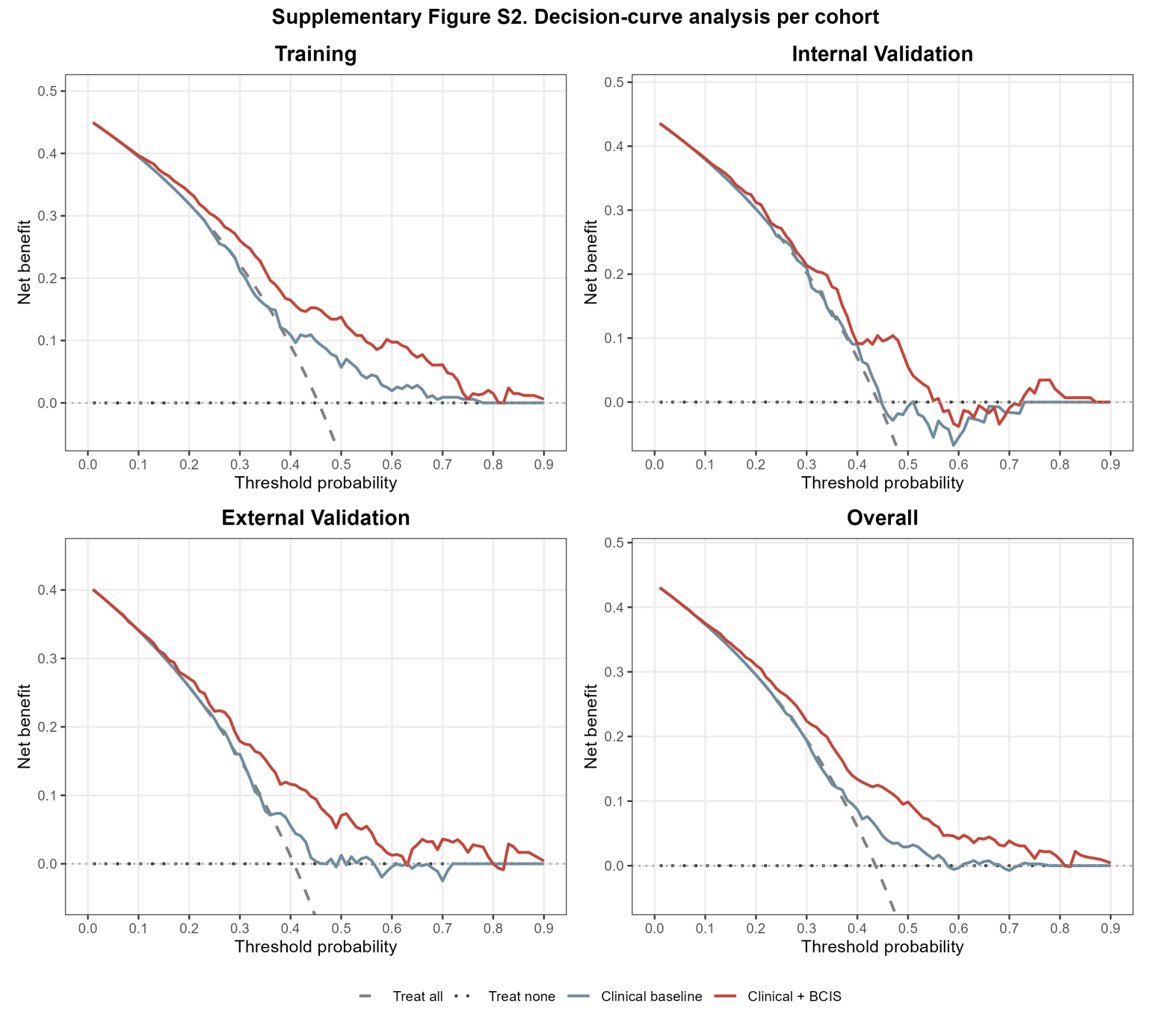
Supplementary Figure S2.**

Decision-curve analysis of four strategies in the training, internal validation, external validation, and overall cohorts. The four strategies are treat all (dashed), treat none (dotted), clinical baseline (blue-gray), and clinical plus BCIS (red). The x-axis is the threshold probability for clinical action; the y-axis is net benefit. The combined model produced higher net benefit than the clinical baseline across the 0.20 to 0.60 threshold range in all four cohorts.

**Supplementary Table S1. Patient screening flow, exclusions, and comparison of included versus excluded patients.**

**Part A. Reasons for exclusion among 1,156 screened patients.**

| **Category** | **n** | **Proportion of screened** | **Notes** |
| --- | --- | --- | --- |
| Total screened | 1156 | - | - |
| Excluded: prior systemic therapy | 78 | 6.7% | - |
| Excluded: active autoimmune disease on steroids | 12 | 1.0% | - |
| Excluded: organ transplant / hereditary lipid disorder | 8 | 0.7% | - |
| Excluded: M1 disease confirmed at any point | 94 | 8.1% | - |
| Excluded: concurrent active malignancy | 21 | 1.8% | - |
| Excluded: missing pretreatment abdominal CT covering L3 | 83 | 7.2% | - |
| Excluded: incomplete laboratory panel | 47 | 4.1% | - |
| Excluded: missing pathology data (TRG / ypT / ypN) | 52 | 4.5% | - |
| Excluded: inadequate follow-up (<6 months without event) | 41 | 3.5% | - |
| Included in analysis | 720 | 62.3% | - |

**Part B. Demographic comparison of included (n=720) versus excluded (n=436) patients.**

| **Characteristic** | **Included (n=720)** | **Excluded (n=436)** | **P value** |
| --- | --- | --- | --- |
| Age, median (IQR) | 61 (56-68) | 63 (55-71) | 0.318 |
| Male sex, n (%) | 539 (74.9%) | 329 (75.5%) | 0.834 |
| Clinical stage IVA, n (%) | 263 (36.5%) | 187 (42.9%) | 0.026 |
| EGJ adenocarcinoma, n (%) | 179 (24.9%) | 102 (23.4%) | 0.564 |
| ECOG PS ≥1, n (%) | 373 (51.8%) | 254 (58.3%) | 0.029 |

P values from Mann–Whitney U for continuous variables and χ² for categorical variables. Modest differences in clinical stage and ECOG distribution are consistent with the eligibility criteria.

**Supplementary Table S2. Interobserver reproducibility (intraclass correlation coefficients, ICC) of CT-derived body composition measurements.**

| **Measurement** | **Reliability metric** | **ICC (95% CI)** |
| --- | --- | --- |
| SMI | Intraclass correlation coefficient (single rater) | 0.972 (0.964-0.978) |
| SMI | Intraclass correlation coefficient (mean of raters) | 0.986 (0.982-0.989) |
| SMD | Intraclass correlation coefficient (single rater) | 0.948 (0.935-0.958) |
| SMD | Intraclass correlation coefficient (mean of raters) | 0.973 (0.966-0.978) |
| VATI | Intraclass correlation coefficient (single rater) | 0.989 (0.986-0.991) |
| VATI | Intraclass correlation coefficient (mean of raters) | 0.994 (0.993-0.995) |
| SATI | Intraclass correlation coefficient (single rater) | 0.987 (0.984-0.990) |
| SATI | Intraclass correlation coefficient (mean of raters) | 0.993 (0.992-0.995) |

Two-way mixed-effects ICC for absolute agreement, calculated on a randomly selected subset of 80 patients whose L3 slices were independently segmented by two board-certified abdominal radiologists. Both readers were blinded to patient identifiers, treatment outcomes, and all clinical or pathological data. All ICCs comfortably exceed the conventional 0.90 threshold for excellent reproducibility.

**Supplementary Table S3. Prespecified cutoff values for each BCIS component, principal literature sources, and clinical interpretation.**

| **Component** | **Cutoff** | **Principal references** | **Clinical interpretation** |
| --- | --- | --- | --- |
| Low SMI | SMI ≤40.8 cm²/m² (men); ≤34.9 cm²/m² (women) | Martin et al. JCO 2013; Caan et al. JAMA Oncol 2018 | Sarcopenia in Asian gastrointestinal cancer cohorts |
| Low SMD | SMD ≤40.0 HU (men); ≤35.0 HU (women) | Caan et al. JAMA Oncol 2018; Aleixo et al. Cancer 2020 | Myosteatosis cutoffs |
| High VATI | VATI ≥60.0 cm²/m² | Doyle et al. J Cachexia 2013; Lin et al. Front Immunol 2022 | Visceral obesity threshold in Asian gastric cancer |
| Low albumin | <38 g/L | Onodera et al. 1984; Caccialanza et al. ESPEN 2015 | Hypoalbuminemia in surgical oncology |
| Low PNI | <47 | Onodera et al. 1984; Hirahara et al. Anticancer Res 2018 | Prognostic Nutritional Index threshold |
| Low prealbumin | <180 mg/L | Chinese Society of Clinical Nutrition 2020; Bharadwaj et al. 2016 | Visceral protein status threshold |
| High NLR | >3 | Templeton et al. JNCI 2014; Bilen et al. Cancer 2019 | Systemic inflammation threshold |
| High CRP | >10 mg/L | Pearson et al. Circulation 2003; Proctor et al. EJC 2011 | Clinical inflammation threshold |
| High CAR | >0.25 | Liu et al. World J Surg Oncol 2015; Saito et al. Anticancer Res 2017 | CRP-to-albumin ratio in gastric cancer |
| Anemia | Hb <120 g/L (men); <110 g/L (women) | WHO 2011 | Pretreatment anemia, WHO definition |

All cutoffs were prespecified before validation analyses and were drawn from peer-reviewed cohorts in Asian gastrointestinal cancer where possible. Sex-specific thresholds for SMI, SMD, and anemia reflect documented sex differences in body composition and hemoglobin distribution.

**Supplementary Table S4. Standardized mean differences before and after inverse probability of treatment weighting (IPTW), and IPTW-weighted BCIS–outcome associations.**

**Part A. SMDs for Unfavorable versus Favorable BCIS (n=417).**

| **Covariate** | **SMD before IPTW** | **SMD after IPTW** |
| --- | --- | --- |
| Age | 0.115 | 0.001 |
| Male | -0.023 | -0.002 |
| ECOG_ge1 | -0.248 | -0.010 |
| Diabetes | 0.062 | 0.003 |
| Hypertension | 0.037 | 0.002 |
| cT4 | -0.022 | -0.012 |
| cN23 | 0.090 | -0.005 |
| EGJ | -0.011 | -0.002 |
| PDL1_ge5 | 0.030 | -0.001 |
| dMMR | 0.137 | 0.015 |
| XELOX_back | -0.048 | -0.011 |

**Part B. SMDs for Intermediate versus Favorable BCIS (n=546).**

| **Covariate** | **SMD before IPTW** | **SMD after IPTW** |
| --- | --- | --- |
| Age | 0.178 | 0.005 |
| Male | -0.034 | -0.000 |
| ECOG_ge1 | -0.195 | -0.007 |
| Diabetes | 0.040 | 0.005 |
| Hypertension | 0.226 | 0.016 |
| cT4 | -0.070 | -0.015 |
| cN23 | 0.079 | 0.007 |
| EGJ | -0.075 | 0.000 |
| PDL1_ge5 | 0.085 | 0.004 |
| dMMR | 0.023 | 0.002 |
| XELOX_back | -0.026 | 0.003 |

**Part C. IPTW-weighted multivariable association between BCIS total score and outcomes (n=720).**

| **Outcome** | **IPTW-weighted adjusted OR** | **95% CI** | **P value** |
| --- | --- | --- | --- |
| MPR | 0.61 | 0.56-0.66 | <0.001 |
| pCR | 0.55 | 0.49-0.62 | <0.001 |

Propensity scores estimated from a logistic model including age, sex, ECOG ≥1, diabetes, hypertension, cT4, cN2/N3, EGJ origin, PD-L1 CPS ≥5, MMR status, and chemotherapy backbone. All 11 covariates achieved |SMD|<0.10 after weighting.

**Supplementary Table S5. Individual contribution of each BCIS component to MPR.**

**Part A. Per-component univariable and adjusted association with MPR (n=720).**

| **Component** | **Prevalence** | **Univ OR (95% CI)** | **Univ P** | **Univ AUC** | **Adj OR (95% CI)** | **Adj P** |
| --- | --- | --- | --- | --- | --- | --- |
| Low SMI | 26.1% | 0.51 (0.36-0.73) | <0.001 | 0.562 | 0.52 (0.36-0.74) | <0.001 |
| Low SMD | 47.5% | 0.73 (0.54-0.98) | 0.033 | 0.540 | 0.69 (0.51-0.94) | 0.020 |
| High VATI | 12.6% | 0.97 (0.62-1.51) | 0.877 | 0.502 | 0.96 (0.61-1.51) | 0.844 |
| Low Albumin | 39.7% | 0.50 (0.37-0.68) | <0.001 | 0.581 | 0.46 (0.33-0.63) | <0.001 |
| Low PNI | 51.1% | 0.66 (0.49-0.88) | 0.006 | 0.552 | 0.62 (0.45-0.84) | 0.002 |
| Low Prealbumin | 38.1% | 0.69 (0.51-0.93) | 0.017 | 0.544 | 0.64 (0.47-0.88) | 0.006 |
| High NLR | 45.6% | 0.65 (0.48-0.87) | 0.004 | 0.554 | 0.65 (0.48-0.88) | 0.006 |
| High CRP | 22.5% | 0.41 (0.28-0.61) | <0.001 | 0.572 | 0.40 (0.27-0.59) | <0.001 |
| High CAR | 23.1% | 0.38 (0.26-0.56) | <0.001 | 0.580 | 0.36 (0.24-0.53) | <0.001 |
| Anemia | 23.9% | 0.44 (0.30-0.64) | <0.001 | 0.571 | 0.41 (0.28-0.61) | <0.001 |

**Part B. Leave-one-component-out analysis (BCIS recalculated with the named component removed).**

| **BCIS variant** | **Adjusted OR (95% CI)** | **P value** | **AUC** |
| --- | --- | --- | --- |
| BCIS minus Low SMI | 0.63 (0.56-0.70) | <0.001 | 0.672 |
| BCIS minus Low SMD | 0.61 (0.55-0.68) | <0.001 | 0.681 |
| BCIS minus High VATI | 0.61 (0.54-0.68) | <0.001 | 0.689 |
| BCIS minus Low Albumin | 0.60 (0.53-0.67) | <0.001 | 0.679 |
| BCIS minus Low PNI | 0.56 (0.50-0.64) | <0.001 | 0.694 |
| BCIS minus Low Prealbumin | 0.60 (0.54-0.68) | <0.001 | 0.686 |
| BCIS minus High NLR | 0.61 (0.54-0.68) | <0.001 | 0.680 |
| BCIS minus High CRP | 0.60 (0.53-0.67) | <0.001 | 0.680 |
| BCIS minus High CAR | 0.60 (0.53-0.68) | <0.001 | 0.679 |
| BCIS minus Anemia | 0.64 (0.57-0.71) | <0.001 | 0.671 |

Full 10-component BCIS reference AUC = 0.688, adjusted OR=0.61 (95% CI 0.55 to 0.68). Adjusted analyses control for age, sex, ECOG, cT4, cN2/N3, EGJ origin, PD-L1 CPS ≥5, MMR status, and chemotherapy backbone.

**Supplementary Table S6. Equal-weight BCIS versus LASSO-penalized regression-weighted alternative.**

**Part A. AUC comparison by cohort.**

| **Cohort** | **Equal-weight BCIS AUC** | **LASSO-weighted score AUC** | **Difference (LASSO minus Equal)** |
| --- | --- | --- | --- |
| Training | 0.696 | 0.727 | +0.031 |
| Internal Validation | 0.644 | 0.654 | +0.010 |
| External Validation | 0.702 | 0.672 | -0.030 |
| Overall | 0.688 | 0.696 | +0.008 |

**Part B. LASSO-penalized component coefficients (training cohort, n=334).**

| **Component** | **LASSO coefficient** |
| --- | --- |
| Low SMI | -0.764 |
| Low SMD | -0.350 |
| High VATI | 0.444 |
| Low Albumin | -0.894 |
| Low PNI | -0.080 |
| Low Prealbumin | -0.644 |
| High NLR | -0.298 |
| High CRP | -0.019 |
| High CAR | -0.956 |
| Anemia | -0.914 |

LASSO logistic regression with 5-fold internal cross-validation on the training cohort. Overall AUC differed by only 0.008 between equal-weight and LASSO, and equal-weight performed slightly better in external validation, supporting retention of the equal-weight specification.

**Supplementary Table S7. Redundancy-removed BCIS variants: sensitivity analyses excluding mathematically overlapping components.**

| **BCIS variant** | **Number of components** | **Adjusted OR (95% CI)** | **P value** |
| --- | --- | --- | --- |
| Without Albumin (keep PNI) | 9 | 0.60 (0.53-0.67) | <0.001 |
| Without PNI (keep Albumin) | 9 | 0.56 (0.50-0.64) | <0.001 |
| Without CRP (keep CAR) | 9 | 0.60 (0.53-0.67) | <0.001 |
| Without CAR (keep CRP) | 9 | 0.60 (0.53-0.68) | <0.001 |
| Without Albumin AND CRP | 8 | 0.57 (0.50-0.66) | <0.001 |
| Without PNI AND CAR | 8 | 0.53 (0.46-0.61) | <0.001 |

All variants adjusted for age, sex, ECOG, cT4, cN2/N3, EGJ origin, PD-L1 CPS ≥5, MMR status, and chemotherapy backbone. The closely related pairs are albumin / PNI (PNI = albumin + 5·lymphocyte) and CRP / CAR (CAR = CRP/albumin). All six redundancy-removed variants yielded adjusted ORs essentially indistinguishable from the primary 10-component OR of 0.61.

**Supplementary Table S8. Center-specific external validation across the three external hospitals.**

| **Center** | **n** | **MPR rate** | **Baseline AUC** | **Combined AUC** | **Cal-in-large** | **Cal slope** | **BCIS adj OR (95% CI)** | **P value** |
| --- | --- | --- | --- | --- | --- | --- | --- | --- |
| Baoding Central Hospital | 91 | 33.0% | 0.527 | 0.689 | -0.467 | 0.837 | 0.50 (0.33-0.75) | <0.001 |
| Hengshui People's Hospital | 80 | 43.8% | 0.562 | 0.696 | +0.103 | 0.743 | 0.54 (0.37-0.81) | 0.003 |
| Shijiazhuang People's Hospital | 70 | 47.1% | 0.694 | 0.725 | +0.036 | 0.907 | 0.55 (0.35-0.87) | 0.010 |

Center-specific multivariable logistic regression of MPR on BCIS total score, adjusted for available baseline covariates with non-zero variance within the center. Combined model = clinical baseline + BCIS. All three centers showed combined AUC ≥0.689 and BCIS adjusted ORs tightly clustered between 0.50 and 0.55, supporting cross-center transportability.

**Supplementary Table S9. Adjusted logistic regression for immune-related adverse events and postoperative complications per one-point BCIS increment.**

| **Endpoint** | **Adjusted OR (95% CI)** | **P value** |
| --- | --- | --- |
| Any irAE | 1.12 (1.02-1.22) | 0.017 |
| Grade3plus irAE | 1.08 (0.92-1.28) | 0.344 |
| Postoperative Complication CD2plus | 1.09 (0.98-1.22) | 0.113 |

Adjusted for age, sex, ECOG ≥1, diabetes, hypertension, and chemotherapy backbone (XELOX versus SOX). The BCIS-toxicity association reaches statistical significance only for any-grade irAEs, supporting the interpretation that BCIS-related host vulnerability is reflected primarily in mild-to-moderate immunologic toxicity rather than in severe or treatment-limiting events.

**Supplementary Table S10. Cox proportional-hazards models for PFS and OS with and without pCR adjustment; proportional-hazards (PH) assumption test for BCIS.**

| **Endpoint** | **Specification** | **Adjusted HR (95% CI)** | **P value** | **Schoenfeld PH P** |
| --- | --- | --- | --- | --- |
| PFS | Without pCR adjustment | 1.47 (1.39-1.56) | <0.001 | 0.406 |
| PFS | With pCR adjustment | 1.46 (1.37-1.55) | <0.001 | 0.418 |
| OS | Without pCR adjustment | 1.33 (1.26-1.42) | <0.001 | 0.015 |
| OS | With pCR adjustment | 1.31 (1.24-1.40) | <0.001 | 0.018 |

Both specifications adjust for age, sex, ECOG ≥1, cT4, cN2/N3, EGJ origin, PD-L1 CPS ≥5, MMR status, and chemotherapy backbone. The "with pCR adjustment" model additionally includes pCR as a covariate. The PH assumption was satisfied for PFS but mildly violated for OS (P=0.015), most likely reflecting the prolonged follow-up tail.

**Supplementary Table S11. Subgroup analyses by PD-1 inhibitor type and chemotherapy backbone, with adjustment for the number of neoadjuvant cycles.**

| **Subgroup** | **n** | **Adjusted OR (95% CI)** | **P value** |
| --- | --- | --- | --- |
| PD-1 agent: Camrelizumab | 187 | 0.54 (0.42-0.69) | <0.001 |
| PD-1 agent: Sintilimab | 178 | 0.68 (0.55-0.85) | <0.001 |
| PD-1 agent: Tislelizumab | 146 | 0.48 (0.36-0.65) | <0.001 |
| PD-1 agent: Toripalimab | 118 | 0.66 (0.50-0.86) | 0.003 |
| PD-1 agent: Pembrolizumab | 55 | 0.54 (0.32-0.89) | 0.016 |
| PD-1 agent: Nivolumab | 36 | NA | NA |
| Backbone: SOX (cycles-adjusted) | 467 | 0.58 (0.50-0.66) | <0.001 |
| Backbone: XELOX (cycles-adjusted) | 253 | 0.68 (0.57-0.81) | <0.001 |
| Full cohort, cycles-adjusted (primary) | 720 | 0.61 (0.55-0.68) | <0.001 |
| P-interaction (SOX vs XELOX) | - | - | 0.113 |

Each subgroup was analyzed by an independent multivariable logistic regression of MPR on BCIS total score, adjusting for age, sex, ECOG ≥1, cT4, cN2/N3, EGJ origin, PD-L1 CPS ≥5, MMR status, chemotherapy backbone (where applicable), and number of neoadjuvant cycles. Nivolumab (n=36) was too small to yield a stable estimate. P-interaction between BCIS and chemotherapy backbone (SOX versus XELOX) = 0.113.

**Supplementary Table S12. Additional subgroup analyses of the BCIS–MPR association by ECOG, cT stage, age ≥70 years, and inflammatory burden.**

| **Subgroup** | **n** | **Adjusted OR (95% CI)** | **P value** |
| --- | --- | --- | --- |
| ECOG PS 0 | 347 | 0.61 (0.52-0.72) | <0.001 |
| ECOG PS 1 | 314 | 0.64 (0.54-0.76) | <0.001 |
| ECOG PS ≥2 | 59 | 0.34 (0.18-0.65) | 0.001 |
| cT2-3 | 377 | 0.60 (0.52-0.70) | <0.001 |
| cT4 | 343 | 0.61 (0.52-0.72) | <0.001 |
| Age ≥70 years | 128 | 0.66 (0.49-0.88) | 0.005 |
| Age <70 years | 592 | 0.60 (0.53-0.68) | <0.001 |
| High inflammation (CRP>10 or NLR>3) | 416 | 0.59 (0.50-0.69) | <0.001 |
| Low inflammation | 304 | 0.64 (0.53-0.77) | <0.001 |

High inflammation defined as CRP >10 mg/L OR NLR >3. Each subgroup analyzed by an independent multivariable logistic regression adjusting for the same covariates as the primary analysis. The numerically strongest effect was seen in the small ECOG ≥2 subgroup; consistency across cT and inflammation strata indicates that BCIS is not merely a proxy for tumor burden or inflammation alone.

**Supplementary Table S13. Reclassification metrics and calibration for adding BCIS to the clinical baseline model.**

**Part A. Continuous net reclassification improvement (NRI) and integrated discrimination improvement (IDI), by cohort.**

| **Cohort** | **Total NRI** | **Event NRI** | **Non-event NRI** | **Total IDI** | **Event IDI** | **Non-event IDI** |
| --- | --- | --- | --- | --- | --- | --- |
| Training | +0.551 | +0.342 | +0.209 | +0.1236 | +0.0673 | +0.0562 |
| Internal Validation | +0.299 | +0.188 | +0.111 | +0.0884 | +0.0476 | +0.0408 |
| External Validation | +0.572 | +0.286 | +0.287 | +0.1159 | +0.0473 | +0.0687 |
| Overall | +0.510 | +0.293 | +0.217 | +0.1146 | +0.0571 | +0.0575 |

**Part B. Calibration metrics by cohort and model.**

| **Cohort** | **Model** | **Calibration-in-the-large** | **Calibration slope** | **Brier score** |
| --- | --- | --- | --- | --- |
| Training | Clinical baseline | +0.000 | 1.000 | 0.235 |
| Training | Clinical + BCIS | -0.000 | 1.000 | 0.206 |
| Internal Validation | Clinical baseline | -0.050 | 0.298 | 0.251 |
| Internal Validation | Clinical + BCIS | -0.047 | 0.737 | 0.230 |
| External Validation | Clinical baseline | -0.202 | 0.693 | 0.239 |
| External Validation | Clinical + BCIS | -0.127 | 0.825 | 0.212 |
| Overall | Clinical baseline | -0.077 | 0.754 | 0.240 |
| Overall | Clinical + BCIS | -0.051 | 0.893 | 0.213 |

NRI and IDI quantify the added predictive value of incorporating the BCIS total score into a clinical baseline that already contains cT, cN, age, ECOG, EGJ origin, PD-L1 CPS, MMR status, and chemotherapy regimen. Positive total NRI and IDI indicate net improvement; both event and non-event components are positive in every cohort.

**Supplementary Table S14. Cohort-stratified decision-curve net benefit at four prespecified threshold probabilities.**

| **Cohort** | **Threshold** | **NB clinical baseline** | **NB clinical + BCIS** | **Δ NB (combined minus baseline)** | **NB treat-all** |
| --- | --- | --- | --- | --- | --- |
| Training | 0.20 | +0.319 | +0.338 | +0.019 | +0.319 |
| Training | 0.30 | +0.212 | +0.260 | +0.049 | +0.222 |
| Training | 0.40 | +0.109 | +0.165 | +0.056 | +0.092 |
| Training | 0.50 | +0.057 | +0.138 | +0.081 | -0.090 |
| Internal Validation | 0.20 | +0.302 | +0.312 | +0.010 | +0.302 |
| Internal Validation | 0.30 | +0.210 | +0.214 | +0.004 | +0.202 |
| Internal Validation | 0.40 | +0.090 | +0.092 | +0.002 | +0.069 |
| Internal Validation | 0.50 | -0.007 | +0.055 | +0.062 | -0.117 |
| External Validation | 0.20 | +0.258 | +0.271 | +0.012 | +0.258 |
| External Validation | 0.30 | +0.160 | +0.179 | +0.019 | +0.152 |
| External Validation | 0.40 | +0.055 | +0.116 | +0.061 | +0.011 |
| External Validation | 0.50 | +0.012 | +0.071 | +0.058 | -0.187 |
| Overall | 0.20 | +0.295 | +0.310 | +0.015 | +0.295 |
| Overall | 0.30 | +0.194 | +0.224 | +0.030 | +0.194 |
| Overall | 0.40 | +0.087 | +0.134 | +0.047 | +0.060 |
| Overall | 0.50 | +0.029 | +0.099 | +0.069 | -0.128 |

Net benefit = (true positives / N) minus (false positives / N) times [threshold / (1 minus threshold)]. Across all four cohorts and all four prespecified thresholds, the combined model produced higher net benefit than the clinical baseline alone. Threshold 0.20 to 0.30 corresponds to a low-cost decision (prehabilitation referral); 0.30 to 0.40 to treatment intensification; 0.40 to 0.50 to consideration of regimen alternatives.
